# Overcoming resistance to anti-PD-1/PD-L1 therapy in cancer

**DOI:** 10.20517/cdr.2026.12

**Published:** 2026-05-15

**Authors:** Lijun Li, Yanbin Zhao, Xinhong Shi, Xiaotian Guo, Xiaoxin Zhang, Guangrui Li, Qingwei Meng, Minghui Zhang, Mingzhu Yin

**Affiliations:** ^1^Clinical Research Center (CRC), Chongqing University Three Gorges Hospital, Chongqing University, Chongqing 404100, China.; ^2^School of Medicine Chongqing University, Chongqing University, Chongqing 400030, China.; ^3^Department of Medical Oncology, Harbin Medical University Cancer Hospital, Harbin 150081, Heilongjiang, China.; ^4^Clinical Research Center (CRC), Medical Pathology Center (MPC), Cancer Early Detection and Treatment Center (CEDTC) and Translational Medicine Research Center (TMRC), Chongqing University Three Gorges Hospital, Chongqing University, Chongqing 404100, China.; ^#^Authors contributed equally.

**Keywords:** Programmed cell death protein 1, programmed cell death ligand 1, drug resistance

## Abstract

Recently, programmed death-1 (PD-1)/programmed death-ligand 1 (PD-L1) inhibitors have achieved major breakthroughs in oncology, with 32 drugs approved over the past decade. This advancement has established immunotherapy as the fifth major antitumor modality following surgery, chemotherapy, radiotherapy, and targeted therapy. However, PD-1/PD-L1 inhibitors induce sustained responses in only a limited number of patients, and primary and acquired resistance remain critical challenges in clinical practice. As understanding of the complex crosstalk among cancer cells, the tumor microenvironment, and the host immune system deepens, numerous strategies to overcome PD-1/PD-L1 inhibitor resistance have been proposed. In this review, we examine the current development of PD-1/PD-L1 inhibitors, analyze global approval trends, and evaluate their monotherapy efficacy across various tumor types. As multi-target combination therapy is an essential strategy for overcoming resistance, we analyze key combination targets - such as vascular endothelial growth factor (VEGF), cytotoxic T-lymphocyte-associated protein 4 (CTLA-4), and lymphocyte activation gene 3 (LAG-3) - and highlight the clinical success of novel dual-target regimens such as ivonescimab (PD-1/VEGF). Furthermore, we discuss potential approaches to overcoming resistance from both microenvironmental (e.g., targeting cancer-associated fibroblasts or utilizing antibody-drug conjugates) and macroenvironmental (e.g., modulating the microbiota or sex hormones) perspectives. This review provides a forward-looking framework for designing precision- and mechanism-driven combination therapies aimed at converting non-responders into responders.

## INTRODUCTION

**Figure 1:**
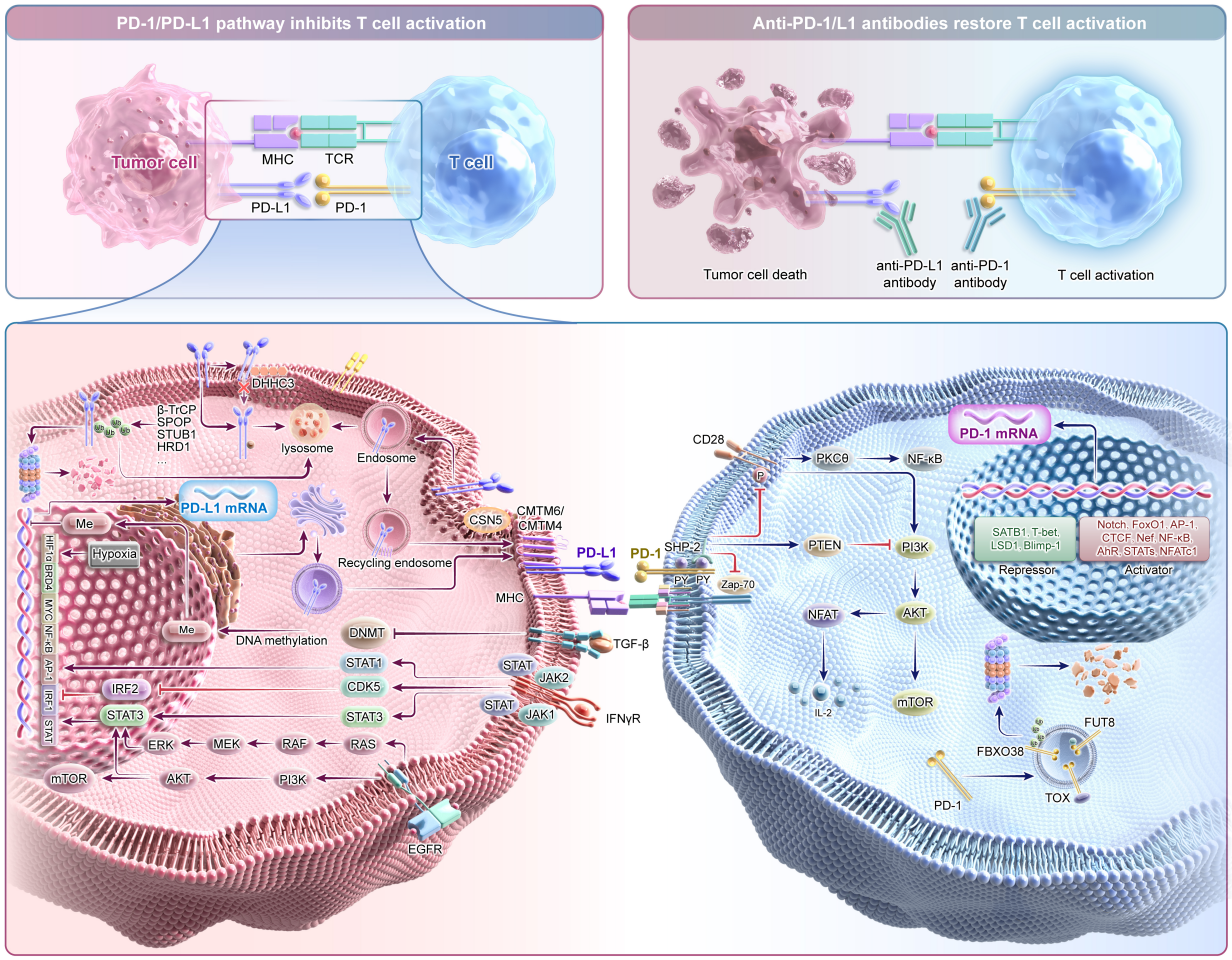
Regulatory network of the PD-1/PD-L1 signaling pathway. The interaction between PD-1 and PD-L1 inhibits TCR signaling, thereby suppressing T cell activation. Expression of this axis is modulated through diverse mechanisms, ranging from genomic alterations to targeted protein degradation. Blockade of the PD-1/PD-L1 axis with anti-PD-1/PD-L1 antibodies can relieve T cell suppression and promote tumor cell eradication. PD-1: Programmed death-1; PD-L1: programmed death-ligand 1; TCR: T cell receptor; MHC: major histocompatibility complex; DHHC3: palmitoyltransferase DHHC3; Ub: Ubiquitin; β-TrCP: beta-transducin repeat containing protein; SPOP: speckle-type POZ protein; STUB1: STIP1 homology and U-box containing protein 1; HRD1: HMG-CoA reductase degradation protein 1; CSN5: COP9 signalosome 5; CMTM: CKLF-like MARVEL transmembrane domain containing; TGF-β: transforming growth factor beta; DNMT: DNA methyltransferase; Me: methylation; IFNγR: interferon gamma receptor; JAK: Janus kinase; STAT: signal transducer and activator of transcription; CDK5: cyclin dependent kinase 5; IRF: interferon regulatory factor; EGFR: epidermal growth factor receptor; RAS: rat sarcoma virus oncogene homolog; RAF: RAF proto-oncogene serine/threonine-protein kinase; MEK: mitogen-activated protein kinase kinase; ERK: extracellular signal-regulated kinase; PI3K: phosphoinositide 3-kinase; AKT: AKT serine/threonine kinase; mTOR: mechanistic target of rapamycin; HIF-1α: hypoxia-inducible factor 1 subunit alpha; BRD4: bromodomain containing 4; MYC: MYC proto-oncogene; NF-κB: nuclear factor kappa B; AP-1: activator protein 1; SHP-2: SH2 domain-containing protein tyrosine phosphatase 2; Zap-70: zeta-chain-associated protein kinase 70; PKCθ: protein kinase C theta; PTEN: phosphatase and tensin homolog; NFAT: nuclear factor of activated T cells; IL-2: interleukin-2; SATB1: special AT-rich sequence-binding protein 1; T-bet: T-box transcription factor 21; LSD1: lysine specific demethylase 1; Blimp-1: B lymphocyte-induced maturation protein-1; FoxO1: forkhead box O1; CTCF: CCCTC-binding factor; AhR: aryl hydrocarbon receptor; NFATc1: nuclear factor of activated T cells 1; TOX: thymocyte selection-associated high mobility group box protein; FUT8: fucosyltransferase 8; FBXO38: F-box protein 38.

Immune checkpoint inhibitors (ICIs), particularly programmed death-1 (PD-1)/programmed death-ligand 1 (PD-L1) monoclonal antibodies, have markedly improved survival outcomes for cancer patients. Research on PD-1/PD-L1 began in 1992, when Ishida *et al.* cloned the complementary DNA (cDNA) of PD-1 from immune cell lines undergoing apoptosis^[1]^. Subsequently, a series of studies investigating the function and therapeutic potential of PD-1/PD-L1 emerged [Supplementary Figure 1]. Through backcrossing PD-1 knockout (KO) heterozygous mice with inbred lines and immunodeficient strains, Nishimura demonstrated how the absence of this receptor drives the activation of T lymphocytes^[2]^. In 1999, Dong *et al.* first identified B7 homolog 1 (B7-H1) by screening human cDNA libraries for homologous sequences and clarified its regulatory role in T cells^[3]^. Later, the interaction between PD-1 and B7-H1 (also known as PD-L1) was rapidly recognized^[4]^. The path from discovery to clinical translation has been extensively explored. An early obstacle was the discrepancy between laboratory and clinical samples: fresh tumor biopsies frequently displayed significant PD-L1 levels, whereas cancer cells grown *in vitro* typically lacked this ligand. In 2002, Dong *et al.* demonstrated that PD-L1 overexpression in tumors is primarily regulated by interferon gamma (IFN-γ)^[5]^. Most importantly, the study showed that anti-PD-L1 antibodies could restore T cell function and inhibit tumor growth in preclinical models. Subsequent studies by Iwai *et al.* and Curiel *et al.* further supported that blocking the interaction between PD-1 and its ligand could effectively suppress cancers^[6,7]^. Collectively, these findings underscored the critical role of the PD-1/PD-L1 pathway in tumor immune evasion and stimulated further investigation into its regulatory mechanisms, ranging from genomic alterations to protein-level modifications [Figure 1]. The transformative impact of this foundational research was recognized in 2018 with the Nobel Prize awarded to Prof. Honjo.

Since the first PD-1 inhibitor received U.S. Food and Drug Administration (FDA) approval in 2014, clinical applications of PD-1/PD-L1 blockade have expanded significantly across oncology. Compared with traditional treatment, PD-1/PD-L1 inhibitors have achieved higher disease control rates and improved overall survival (OS). However, poor responses are still observed in certain patient groups, and durable responses occur only in a subset of patients, even among tumors typically sensitive to ICIs. Therefore, primary or secondary resistance remains a major clinical challenge. As research continues to explore the multidimensional crosstalk among tumors, the immune system, and other biological systems, our comprehension of anti-PD-1/PD-L1 resistance mechanisms continues to evolve^[8]^. Tumor-intrinsic components, including tumor cells and their secretions, along with immune cells, stromal cells, and the microbiome, collectively serve as key determinants of therapeutic response to anti-PD-1/PD-L1 therapy. Tumor-intrinsic factors form the foundation of immune responses, while the tumor immune microenvironment shapes the overall immune landscape. Additionally, systemic host-related factors such as sex, obesity, and microbiota composition may also affect responses to anti-PD-1/PD-L1 therapy^[9-11]^. Beyond host-intrinsic influences, the external environment may further modulate cancer biology and potentially contribute to therapeutic resistance. Given that anti-tumor immunity is intricately regulated by both micro- and macro-environment factors, combination therapy may be an essential strategy to maximize the therapeutic potential of PD-1/PD-L1 blockade.

This article summarizes globally approved PD-1/PD-L1 inhibitors and outlines the current therapeutic landscape. We first analyzed objective response rates (ORRs) from 118 clinical trials, which included a total of 22,433 patients across different cancer types. To explore combination strategies for overcoming resistance, we retrieved and analyzed 7,381 PD-1/PD-L1-related clinical trials from ClinicalTrials.gov. Furthermore, this review synthesizes preclinical evidence underlying tumor immune escape during anti-PD-1/PD-L1 therapy, as well as actionable approaches to restoring therapeutic sensitivity.

## APPROVAL OF ANTI-PD-1/PD-L1 DRUGS

**Figure 2:**
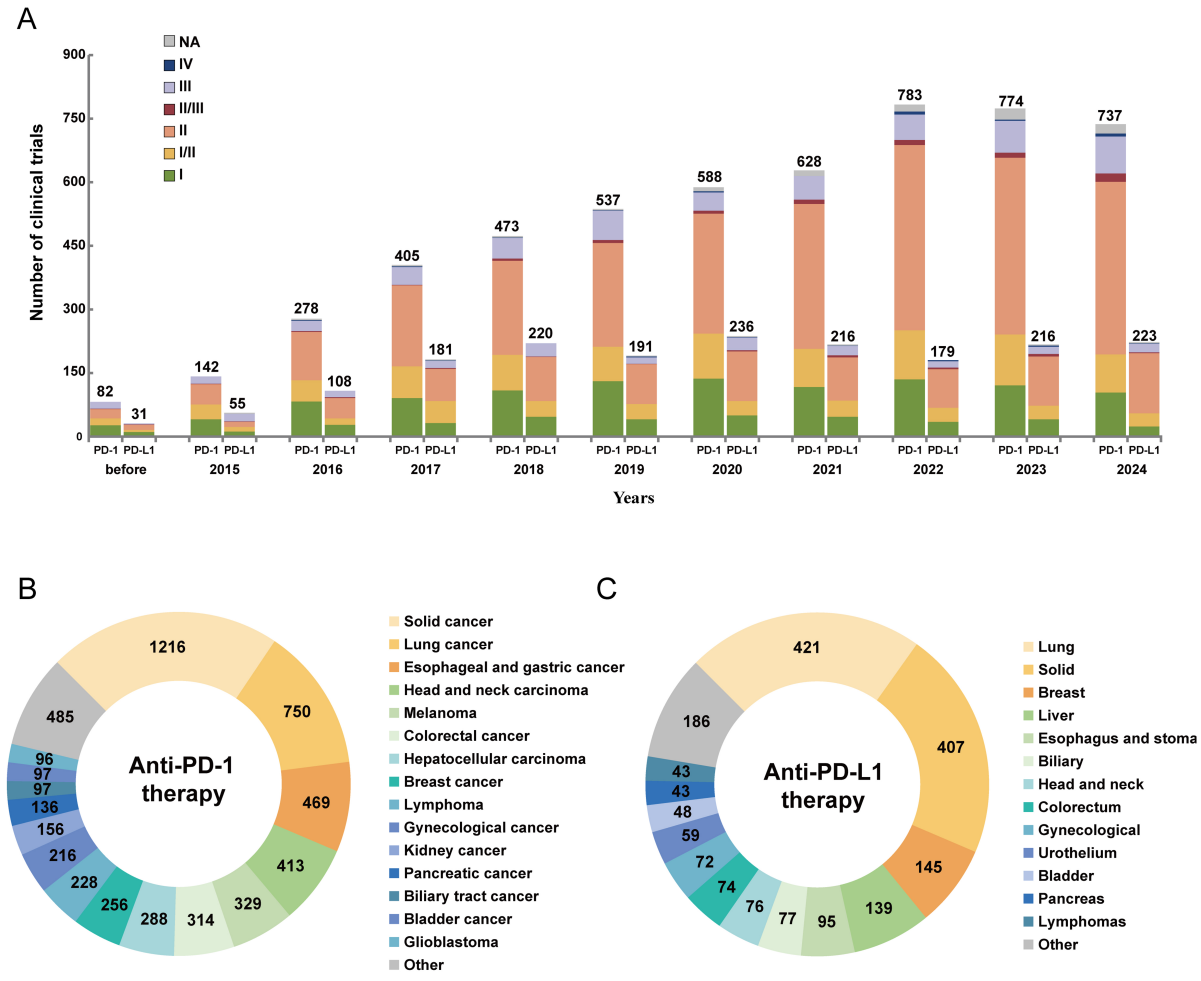
The development landscape of clinical trials related to PD-1/PD-L1 inhibitors. (A) Number of clinical trials involving PD-1/PD-L1 inhibitors across different phases; (B) Distribution of cancer types in clinical trials related to anti-PD-1 therapy; (C) Distribution of cancer types in clinical trials related to anti-PD-L1 therapy. Data from ClinicalTrials.gov. PD-1: Programmed death-1; PD-L1: programmed death-ligand 1; NA: not applicable.

**Figure 3:**
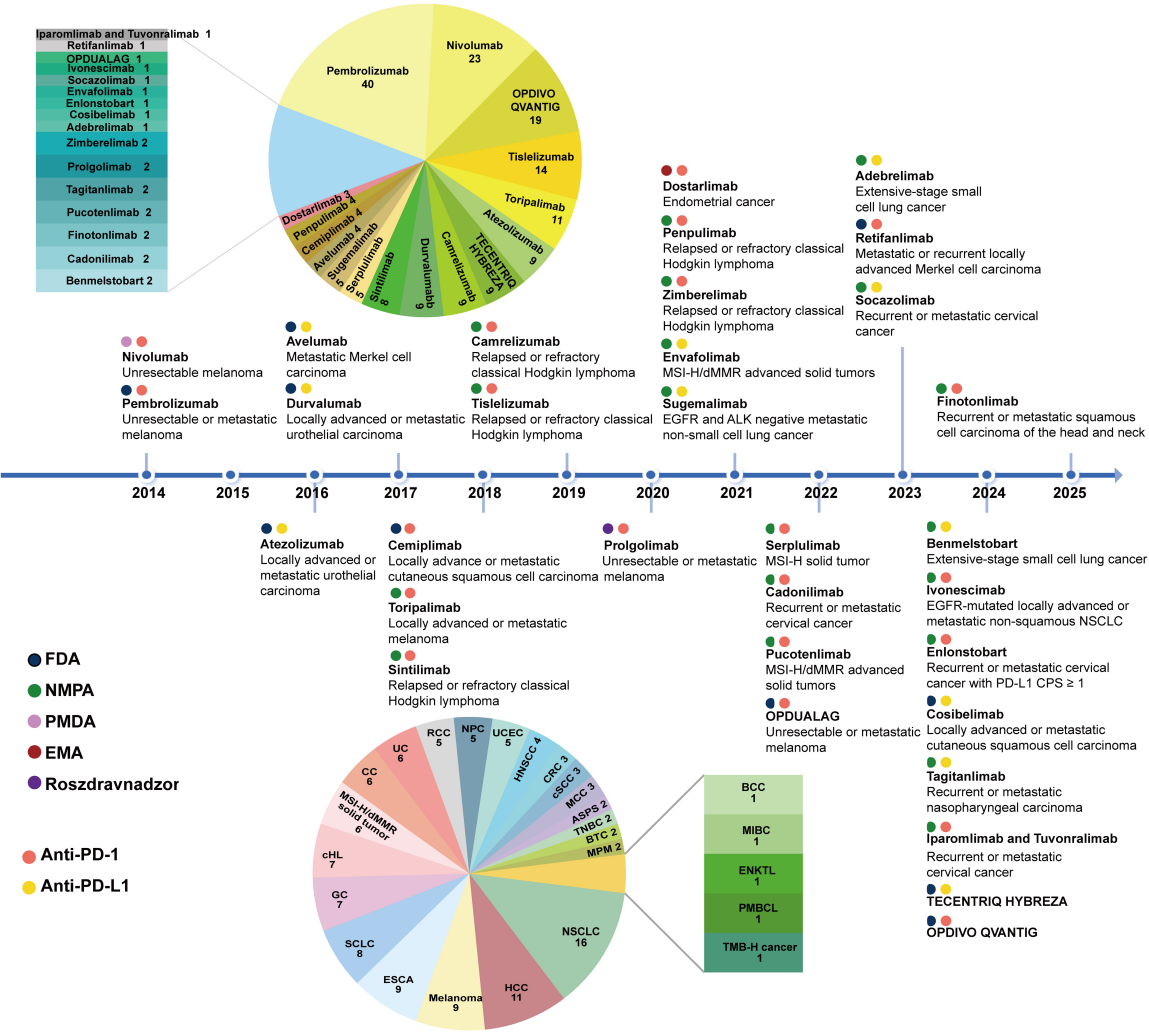
Timeline of PD-1/PD-L1 inhibitor approvals. The timeline illustrates the first regulatory agency to approve a PD-1/PD-L1 inhibitor and the indications for 32 PD-1/PD-L1 inhibitors. The pie chart in the upper left shows the number of approved indications for each drug. The pie chart in the lower left displays the number of available PD-1/PD-L1 inhibitors for each cancer type. PD-1: Programmed death-1; PD-L1: programmed death-ligand 1; MSI-H: microsatellite instability-high; dMMR: mismatch repair deficiency; EGFR: epidermal growth factor receptor; ALK: anaplastic lymphoma kinase; FDA: Food and Drug Administration; NMPA: National Medical Products Administration (China); PMDA: Pharmaceuticals and Medical Devices Agency (Japan); EMA: European Medicines Agency; Roszdravnadzor: Federal Service for Surveillance in Healthcare (Russia); NSCLC: non-small cell lung cancer; CPS: combined positive score; CC: cervical cancer; UC: urothelial cancer; RCC: renal cell carcinoma; NPC: nasopharyngeal carcinoma; UCEC: endometrial cancer; HNSCC: head and neck squamous cell cancer; CRC: colorectal cancer; cSCC: cutaneous squamous cell carcinoma; MCC: Merkel cell carcinoma; ASPS: alveolar soft part sarcoma; TNBC: triple-negative breast cancer; BTC: biliary tract cancer; MPM: malignant pleural mesothelioma; HCC: hepatocellular carcinoma; ESCA: esophageal cancer; SCLC: small cell lung cancer; GC: gastric cancer; cHL: classical Hodgkin lymphoma; BCC: basal cell carcinoma; MIBC: muscle-invasive bladder cancer; ENKTL: extranodal NK/T cell lymphoma; PMBCL: primary mediastinal large B-cell lymphoma; TMB-H: tumor mutational burden-high.

A comprehensive search of ClinicalTrials.gov identified 7,381 clinical trials evaluating PD-1/PD-L1 blockade by April 2025 [Figure 2]. Globally, 32 PD-1/PD-L1 inhibitors have been approved for antitumor therapy, including 21 targeting PD-1 and 11 targeting PD-L1 [Figure 3]. Among these, 10 drugs were approved by the U.S. FDA, 19 by the Chinese National Medical Products Administration (NMPA), and the remaining three were approved in Japan, Europe, and Russia, respectively. Notably, the pace of development has accelerated markedly since 2021, with 21 drugs approved over the past 4 years. The first bispecific antibody targeting PD-1 and cytotoxic T-lymphocyte-associated protein 4 (CTLA-4), cadonilimab, received approval in 2022^[12]^. Two years later, the first bispecific antibody targeting PD-1 and vascular endothelial growth factor (VEGF), ivonescimab, was also approved^[13]^. Additionally, PD-1/lymphocyte activation gene 3 (LAG-3) or PD-1/CTLA-4 bifunctional combination antibodies (OPDUALAG, iparomlimab, and tuvonralimab) were approved in 2022 and 2024, respectively^[14,15]^.

Malignant melanoma served as the initial clinical focus for PD-1/PD-L1 inhibitors. The therapeutic landscape subsequently expanded to encompass non-small cell lung cancer (NSCLC), nasopharyngeal carcinoma, and other malignancies [Figure 3]. Pembrolizumab has received approval for the widest range of indications, covering 40 indications across 20 cancer types, including malignant melanoma, NSCLC, and head and neck squamous cell carcinoma^[16]^. Atezolizumab is the first approved PD-L1 inhibitor and has nine indications across five cancer types^[17]^. NSCLC has the largest number of approved therapeutic options, including 11 PD-1 inhibitors, four PD-L1 inhibitors, and one PD-1/VEGF bispecific antibody^[18,19]^. This is followed by hepatocellular carcinoma (HCC), malignant melanoma, and esophageal cancer. Similarly, the exploration of indications in clinical trials broadly aligns with this trend [Figure 2B and C]. The drug development landscape corresponds well with tumor epidemiological characteristics, as both NSCLC and HCC rank among the top 10 cancers in incidence and mortality^[20]^. Currently, 32 approved PD-1/PD-L1 inhibitors are available for 26 cancer types, covering not only first- and later-line treatments for advanced disease but also adjuvant and neoadjuvant applications for early disease.

## ANTI-PD-1/PD-L1 MONOTHERAPY EFFICACY AND COMBINATION STRATEGIES

**Figure 4:**
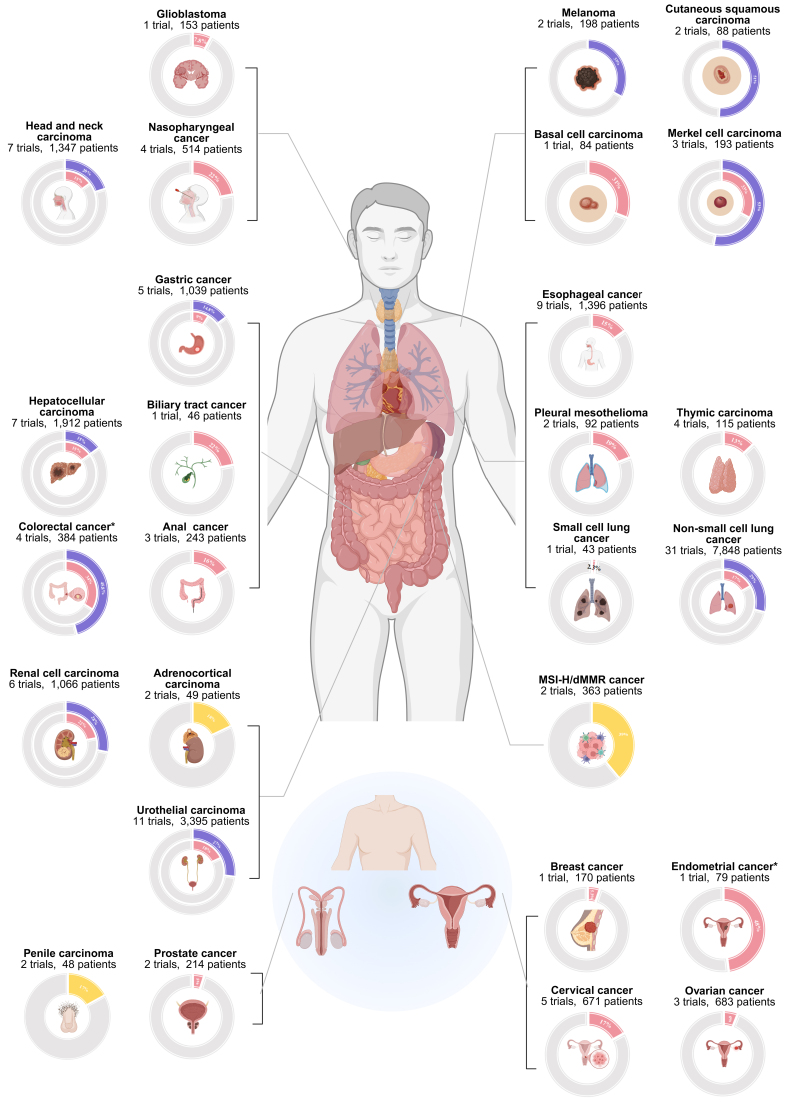
ORRs for PD-1/PD-L1 inhibitors as monotherapy. Purple circles show ORRs for PD-1/PD-L1 inhibitors as first-line treatment; pink circles show ORRs for their use in subsequent therapy; yellow circles show ORRs regardless of treatment line. ^*^Tumors are microsatellite instability-high or mismatch repair-deficient. Created in BioRender. li, L. (2026) https://BioRender.com/kws7r1d. ORRs: Objective response rates; PD-1: programmed death-1; PD-L1: programmed death-ligand 1; MSI-H: microsatellite instability-high; dMMR: mismatch repair deficiency.

Despite their broad application, single-agent PD-1/PD-L1 blockade has shown heterogeneous clinical outcomes across cancer types, and not all patients benefit^[21]^. To evaluate these variable response rates, we conducted a comprehensive literature search in PubMed, which initially retrieved 12,842 records [Supplementary Table 1 and Supplementary Figure 2]. After screening, 118 eligible clinical trials were included, involving 22,433 patients with advanced solid tumors [Figure 4 and Supplementary Table 2]. Overall, the data revealed that first-line administration of these monotherapies achieved ORRs ranging from 14.8% to 53%, whereas responses in later-line settings ranged from 2.3% to 48%. In tumors such as Merkel cell carcinoma, cutaneous squamous carcinoma, and microsatellite instability-high colorectal cancer, ORRs reached about 50% in the first-line setting and 30% in later lines^[22,23]^. However, in gastric and liver cancers, first-line ORRs were only around 15%^[24]^. Improving anti-PD-1/PD-L1 response therefore remains a critical challenge.

**Figure 5:**
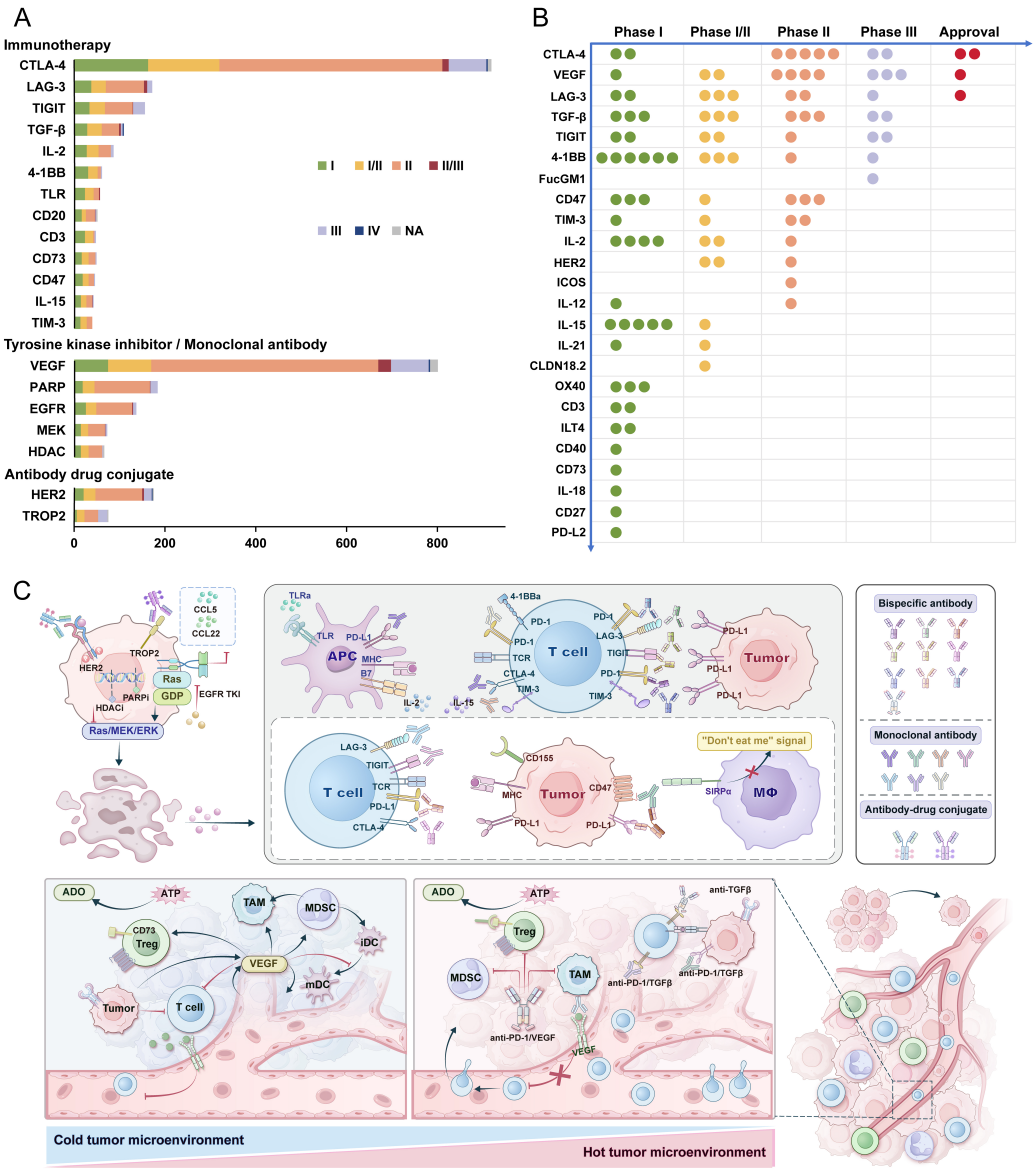
Drug targets combined with PD-1/PD-L1 inhibitors in clinical trials. (A) Top 20 combination therapy targets across 7,381 clinical trials; (B) PD-1/PD-L1-related bispecific or bifunctional combination antibodies in clinical trials or on the market, with each dot representing one drug; (C) Mechanisms of the most common combination therapy targets. PD-1: Programmed death-1; PD-L1: programmed death-ligand 1; CTLA-4: cytotoxic T-lymphocyte-associated protein 4; LAG-3: lymphocyte activation gene 3; TIGIT: T cell immunoreceptor with immunoglobulin and immunoreceptor tyrosine-based inhibitory motif domains; TGF-β: transforming growth factor beta; IL: interleukin; TLR: Toll-like receptor; CD: cluster of differentiation; TIM-3: T cell immunoglobulin and mucin domain-containing protein 3; NA: not available; VEGF: vascular endothelial growth factor; PARP: poly (ADP-ribose) polymerase; EGFR: epidermal growth factor receptor; MEK: mitogen-activated protein kinase kinase; HDAC: histone deacetylase; HER2: human epidermal growth factor receptor 2; TROP2: trophoblast cell surface antigen 2; ICOS: inducible T-cell costimulator; CLDN: claudin; OX40: OX40 molecule; ILT4: immunoglobulin-like transcript 4; PD-L2: programmed death-ligand 2; CCL: C-C motif chemokine ligand; GDP: guanosine diphosphate; TKI: tyrosine kinase inhibitor; ERK: extracellular signal-regulated kinase; APC: antigen-presenting cell; MHC: major histocompatibility complex; TCR: T cell receptor; SIRPα: signal regulatory protein alpha; ADO: adenosine; ATP: adenosine triphosphate; Treg: regulatory T; TAM: tumor-associated macrophage; MDSC: myeloid-derived suppressor cell; iDC: immature dendritic cell; mDC: mature dendritic cell.

Combination therapies targeting multiple pathways to enhance antitumor activity are the main strategy to address this challenge. Analysis of 7,381 PD-1/PD-L1-related clinical trials showed that VEGF and CTLA-4 are the most common combination targets, with other major targets including poly (ADP-ribose) polymerase (PARP), human epidermal growth factor receptor 2 (HER2), LAG-3, and T cell immunoreceptor with immunoglobulin (Ig) and immunoreceptor tyrosine-based inhibitory motif (ITIM) domains (TIGIT) [Figure 5A]. Bispecific antibodies represent a promising direction^[25]^. Beyond the four approved PD-1-related bispecific or bifunctional agents, 12 additional drugs are currently in phase III clinical trials and may be approved in the near future [Figure 5B]. The antitumor mechanisms of these common combination targets are illustrated in Figure 5C. The addition of bevacizumab to anti-PD-1/PD-L1 therapy has been widely used in patients with cancers^[26,27]^. Recently, combinations with other anti-angiogenic agents, such as lenvatinib and axitinib, have also gained approval^[28,29]^. Notably, ivonescimab, a bispecific PD-1/VEGF antibody, demonstrated superior efficacy in a head-to-head comparison with pembrolizumab in the HARMONi-2 study. Interim data showed nearly doubled median progression-free survival (PFS) (11.1 *vs.* 5.8 months)^[30]^. Based on these findings, ivonescimab was approved in 2024 for the treatment of EGFR-mutated locally advanced or metastatic non-squamous NSCLC. Findings from the HARMONi-6 trial, which evaluated ivonescimab plus chemotherapy *vs.* tislelizumab plus chemotherapy in advanced squamous NSCLC, further confirmed its antitumor activity^[31]^. Combining PD-1/PD-L1 inhibitors with CTLA-4 inhibitors is one of the earliest dual immunotherapy strategies. Results from the phase III POSEIDON study showed that adding tremelimumab to durvalumab plus chemotherapy significantly improved OS and PFS in metastatic NSCLC without increasing adverse effects^[32]^. Findings from the CheckMate 9LA trial further supported the antitumor efficacy of this approach^[33]^. Co-targeting LAG-3 and the PD-1/PD-L1 axis represents another effective strategy. A phase II/III trial demonstrated that OPDUALAG, a fixed-dose combination of relatlimab and nivolumab, significantly prolonged survival in patients with advanced melanoma compared with nivolumab monotherapy^[34]^. However, not all combination regimens have shown positive outcomes. The first PD-L1/transforming growth factor beta (TGF-β) bifunctional fusion protein (M7824) entered clinical trials in 2015, but several phase II/III studies were terminated due to failure to meet primary endpoints, and no agents in this category have been approved to date^[35,36]^. Safety concerns have also emerged in certain combinations, such as nivolumab plus abemaciclib, as well as pembrolizumab or nivolumab plus pegilodecakin^[37,38]^. Overall, the exploration of combination strategies still faces substantial challenges, requiring continued preclinical research to clarify mechanisms of antitumor immunity and provide theoretical support for overcoming resistance.

## PRECLINICAL RESEARCH ON PD-1/PD-L1 INHIBITOR-BASED THERAPY

**Figure 6:**
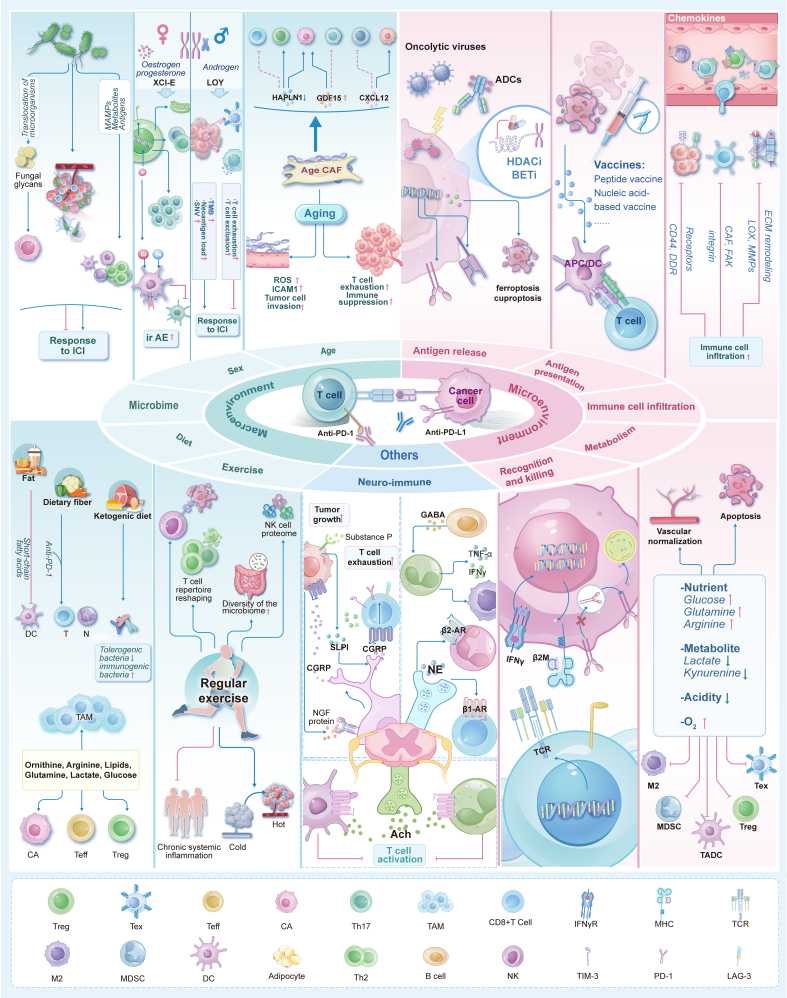
Potential factors influencing PD-1/PD-L1 inhibitor response. Multiple factors within both the macro- and microenvironments interact to regulate antitumor immunity and responses to PD-1/PD-L1 inhibitors. Microenvironmental factors involve elements affecting antigen release, antigen presentation, immune cell activation, and metabolism. Macroenvironmental factors include age, sex, microbiome, diet, and exercise. Recently, the influence of neuroimmune interactions on antitumor immunity has been increasingly recognized, suggesting additional potential targets for overcoming resistance. PD-1: Programmed death-1; PD-L1: programmed death-ligand 1; ADC: antibody-drug conjugate; HDACi: histone deacetylase inhibitor; BETi: bromodomain and extraterminal inhibitor; APC: antigen-presenting cell; DC: dendritic cell; ECM: extracellular matrix; LOX: lysyl oxidase; MMPs: matrix metalloproteinases; CAF: cancer-associated fibroblast; FAK: focal adhesion kinase; DDR: discoidin domain receptor; Tex: exhausted T cell; Treg: regulatory T cell; TADC: tumor-associated dendritic cell; MDSC: myeloid-derived suppressor cell; IFNγ: interferon gamma; β2M: beta-2 microglobulin; TCR: T cell receptor; GABA: gamma-aminobutyric acid; TNF-α: tumor necrosis factor alpha; β1-AR: beta-1 adrenergic receptor; β2-AR: beta-2 adrenergic receptor; NE: norepinephrine; SLPI: secretory leukocyte protease inhibitor; CGRP: calcitonin gene-related peptide; Ach: acetylcholine; NK: natural killer; CA: cancer cell; Teff: effector T cell; TAM: tumor-associated macrophage; N: neutrophil; MAMPs: microbe-associated molecular patterns; ICI: immune checkpoint inhibitor; XCI-E: X-chromosome inactivation escape; LOY: loss of Y chromosome; irAE: immune-related adverse event; TMB: tumor mutational burden; HAPLN1: hyaluronan and proteoglycan link protein 1; GDF15: growth differentiation factor 15; CXCL12: C-X-C motif chemokine ligand 12; ICAM1: intercellular adhesion molecule 1.

To clarify the preclinical landscape of anti-PD-1/PD-L1 resistance, we reviewed the relevant literature from PubMed and summarized potential strategies for restoring drug activity. Disruption at any stage of the cancer-immunity cycle can lead to immune escape, thereby impairing the potency of ICIs. Furthermore, the influence of host-related factors such as sex, age, and gut microbiota is increasingly recognized. This study examined resistance mechanisms from macro- and microenvironmental perspectives, providing theoretical insights into overcoming resistance [Figure 6].

### Promoting antigen release and presentation

The presence of sufficient neoantigens recognizable by the immune system is the first step in initiating antitumor immunity. Enhancing effective antigen release is a key strategy for improving the efficacy of anti-PD-1/PD-L1 inhibitors. Epigenetic drugs represent a classic approach to restoring tumor immunogenicity. However, early clinical trials revealed limited clinical activity when combining histone deacetylase (HDAC) inhibitors with PD-1/PD-L1 inhibitors^[39]^. In addition, novel epigenetic drugs, such as bromodomain and extraterminal (BET) inhibitors, have shown potential for enhancing the efficacy of anti-PD-1/PD-L1 therapies^[40]^. Oncolytic virus therapy is a novel antitumor strategy that enhances immunogenic cell death (ICD) in tumors. Mechanistic studies and early clinical trials have confirmed its role in sensitizing tumors to immunotherapy^[41]^. Recent research has focused on engineering oncolytic viruses that express multiple molecules, such as OX40L, to further enhance the effects of PD-1/PD-L1 inhibitors^[42]^. Antibody-drug conjugates (ADCs) carrying ICD-inducing payloads have also been shown to promote ICD, providing a rationale for combination strategies with immunotherapy. Several clinical studies have preliminarily demonstrated synergistic effects between ADCs and PD-1/PD-L1 inhibitors. Next-generation conjugates, such as bispecific ADCs, stimulator of interferon genes (STING) agonist-loaded ADCs, and conditionally active ADCs, hold promise for enhancing the efficacy of combination therapy while reducing treatment-related adverse events^[43,44]^. Additional methods to promote antigen release are currently being explored in preclinical studies. Antigen release often depends on ICD processes, such as apoptosis, necrosis, and regulated cell death (RCD), including ferroptosis, cuproptosis, pyroptosis, and programmed necrosis. However, most ICD occurs through apoptosis, which is generally immunotolerant and unable to activate antitumor immunity^[45]^. As a result, inducing RCD is emerging as a promising strategy for immunotherapy. Ferroptosis is a major focus in this field. In several mouse tumor models, ferroptosis inducers, such as GPX4 inhibitors and translocator protein (TSPO) inhibitors, have shown strong synergy with anti-PD-1/PD-L1 therapy^[46,47]^. Ferroptosis not only directly suppresses tumor cell proliferation, but also activates dendritic cells (DCs) by releasing damage-associated molecular patterns (DAMPs)^[48]^. Furthermore, induction of ferroptosis by targeting upstream or downstream molecules can also enhance immunotherapy efficacy, as demonstrated by TMOD3 knockdown or heterogeneous nuclear ribonucleoprotein L (HnRNP L) knockdown^[49,50]^. However, ferroptosis-induced immunogenicity is context-dependent^[51]^. Maximizing its therapeutic efficacy requires improving tumor selectivity while reducing the suppressive effects of oxidized lipids on immune cells^[52,53]^. Nanoparticles and similar materials represent a potential solution^[54,55]^. Cuproptosis, proposed in 2022, also shows promise for enhancing anti-PD-1/PD-L1 efficacy, but its clinical application still requires further preclinical research^[56]^.

Tumor antigens are captured by antigen-presenting cells (APCs) and presented to T cells through major histocompatibility complex (MHC) molecules. Reduced numbers of DCs in draining lymph nodes and impaired T cell activation hinder antitumor immunity and limit the efficacy of PD-1/PD-L1 inhibitors. Clinical trials have evaluated antigen vaccines, CD40 agonists, and Toll-like receptor (TLR) agonists to improve responses to anti-PD-1/PD-L1 therapy, but the results have been mixed. A phase I trial showed that the TLR9 agonist vidutolimod plus pembrolizumab induced durable tumor regression in 25% of patients with metastatic melanoma resistant to PD-1 inhibitors^[57]^, whereas a separate phase I study of a TLR7 agonist showed limited benefit either as monotherapy or in combination with a PD-1 inhibitor^[58]^. Preclinical studies are now exploring TLR agonists incorporated into nanomedicines or co-delivery systems to better reverse the suppressive tumor microenvironment (TME) and improve anti-PD-1/PD-L1 activity^[59]^. Neoantigen vaccines activate APCs by directly delivering antigens, and studies have shown that vaccine-induced memory T cells can provide long-term protection^[60]^. Personalized neoantigen vaccines combined with PD-1/PD-L1 blockade have demonstrated preliminary antitumor activity in early-phase trials^[61]^. Current preclinical studies aim to further optimize these combinations by refining neoantigen design, adjusting dosing schedules, and integrating multi-drug strategies. The functional state of DCs also shapes antigen presentation. Sánchez-Paulete *et al.* identified BATF3-dependent DCs as essential for cross-presenting tumor antigens and mediating anti-PD-1 responses^[62]^. The interaction between the STAT3 and STAT5 transcriptional pathways determines DC phenotypes in the TME, and STAT3 degraders represent a potential immunotherapeutic approach^[63]^. Another study found that TP63 and STAT1 mutually inhibit each other and regulate the IFN-γ signaling pathway through co-occupation and co-regulation of promoters and enhancers, and TP63 silencing can enhance PD-1 blockade efficacy^[64]^.

Loss of co-stimulatory factors such as 4-1BB/4-1BBL and CD28/CD80 also impairs T cell activation and contributes to immunotherapy resistance. Combinations of 4-1BB agonists with PD-1/PD-L1 inhibitors, as well as bispecific antibodies, have entered clinical trials. Although the results have not been entirely satisfactory, high response rates have been observed in certain cancers (e.g., melanoma)^[65]^. CD28, another primary co-stimulatory receptor, provides the second activation signal by binding CD80/CD86. Despite early safety concerns, CD28 remains a viable therapeutic target. Majocchi *et al.* recently reported early results for NI-3201, a bispecific antibody that mediates PD-L1-dependent CD28 co-stimulation, and clinical studies are being planned^[66]^. However, another study has identified a distinct function of CD28 in tumor cells - promoting immune evasion. Knockdown of tumor-cell CD28 may overcome anti-PD-1 resistance by affecting PD-L1^[67]^. Because CD28 exerts different functions in tumor cells and T cells, therapeutic targeting requires strict cell specificity. RNA sequencing studies have also identified novel targets, such as mitochondrial antiviral signaling protein (MAVS) and FTSJ3, which modulate antitumor immunity through IFN signaling and may sensitize tumors to PD-1/PD-L1 blockade^[68,69]^. These findings offer new insights into combination strategies, though clinical translation remains ongoing.

### Enhancing the trafficking and infiltration of immune cells

#### Modulating chemokine networks

Chemokines are key cytokines that regulate immune cell migration and lymphoid tissue development and are grouped into four families: C, CC, CXC, and CX3C. Immune cells show distinct migratory responses within the TME based on their chemokine receptor expression profiles. Antitumor immune cells, including CD8^+^ T cells, natural killer (NK) cells, and Th1 cells, mainly rely on CXCR3 and its ligands CXCL9/10, whereas regulatory T (Treg) cells depend on the CCR4-CCL22/28 axis, and myeloid-derived suppressor cells (MDSCs) and tumor-associated macrophages (TAMs) are driven by the CCR2-CCL2 axis^[70]^. Emerging evidence highlights the capacity of CXCL8 to increase macrophage PD-L1 levels by activating purine metabolism^[71]^. Therefore, modulating chemokines to reshape the immunosuppressive TME is a potential strategy to enhance anti-PD-1/PD-L1 activity. Early clinical trials combining CCR4 antagonists (e.g., mogamulizumab) or CXCR4 antagonists (e.g., BL-8040) with PD-1/PD-L1 inhibitors have demonstrated favorable safety profiles and preliminary antitumor activity^[72,73]^. Bergeron *et al.* reported that the addition of a CXCR2 antagonist to partial irradiation and anti-PD-1 therapy reduced irradiation-induced CXCR2-driven neutrophil infiltration and strengthened therapeutic efficacy^[74]^. Recent studies have also shown that regulating chemokine expression by targeting upstream regulators may overcome anti-PD-1/PD-L1 resistance. For instance, inhibition of lysine-specific demethylase 4C (KDM4C) increased CXCL10 transcription through enhanced H3K36me3 occupancy at the promoter region, and the combination of KDM4C inhibition, radiotherapy, and anti-PD-L1 treatment yielded the strongest antitumor activity with manageable toxicity in a lung cancer mouse model^[75]^. WNT11-overexpressing tumor cells limit CD8^+^ T cell recruitment by reducing CXCL10 and CCL4 through CAMKII-mediated β-catenin/AFF3 downregulation, and CAMKII inhibition has shown synergy with anti-PD-1 therapy in liver metastasis models^[76]^. Additional targets involved in chemokine signaling have also been identified, including translationally controlled tumor protein (TCTP), which activates the EGFR-AKT-MCL-1/CXCL10 pathway^[77]^; lymphotoxin beta receptor (LTβR), which induces tertiary lymphoid structure (TLS)-associated chemokines^[78]^; and zinc finger E-box-binding homeobox 1 (ZEB1), which suppresses CXCL10 secretion and restricts CD8^+^ T cell infiltration^[79]^. Although clinical trials focused on chemokine modulation remain limited, existing early clinical trials and preclinical evidence indicate that this strategy warrants continued investigation.

#### Normalizing tumor vasculature

When immune cells reach tumor sites, they undergo a complex process of infiltration and reactivation. Tumor cells promote tumor vascularization through vessel co-option and angiogenesis, which supports tumor growth and facilitates immune evasion. Immune cells in the TME can also stimulate angiogenesis. This bidirectional interaction between tumor vasculature and immune cells drives the development of an immunosuppressive phenotype. Anti-angiogenic therapy is one of the clinically validated strategies that enhance the activity of PD-1/PD-L1 inhibitors, but this regimen has not markedly improved OS in some cancers^[80]^. Tumor hypoxia is a major factor limiting therapeutic efficacy. Studies have shown that prolonged use of anti-angiogenic drugs at approved doses excessively prunes tumor vessels, worsening hypoxia and immune suppression, thereby reducing the effectiveness of immunotherapy^[81]^. Concurrent anti-angiogenic therapy may also reduce the intratumoral distribution of anti-PD-1/PD-L1 antibodies^[82]^. Additionally, the impact of vascular selectivity and “non-angiogenic” tumors is often underestimated. Tumors that rely on vessel co-option are inherently resistant to VEGF inhibitors and often exhibit poor immune cell infiltration^[83]^. Future strategies should move beyond simple vascular blockade toward more precise regulation. Sustaining a prolonged “vascular normalization” window by lowering drug doses and shortening treatment cycles has been shown to improve perfusion and immune cell infiltration^[84]^. Co-inhibition of VEGF and hypoxia-inducible factor-1α (HIF-1α) within the hypoxia pathway promotes vascular normalization and strengthens PD-1/PD-L1 blockade efficacy^[85]^. In addition, multi-target combinations and novel delivery platforms represent emerging approaches to normalizing tumor vasculature and overcoming resistance to PD-1/PD-L1 inhibitors^[86,87]^.

#### Remodeling the extracellular matrix

The extracellular matrix (ECM) is a crucial component of the TME. It serves as a physical barrier that impedes immune cell infiltration and regulates immune cell activity through biochemical signaling. The dual physical and physiological barriers created by ECM remodeling are considered a mechanism of immunotherapy resistance. Cancer-associated fibroblasts (CAFs) are drivers of ECM remodeling and secrete several collagens and cross-linking enzymes to construct a dense fibrotic matrix. Enhancing active immune cell infiltration by targeting CAFs has proven to be an effective approach to potentiating PD-1/PD-L1 blockade^[88,89]^. Lysyl oxidase (LOX) catalyzes collagen cross-linking, thereby increasing matrix stiffness and activating pro-cancer signaling pathways, such as the integrin-FAK-Src pathway^[90]^. In addition, this process increases interstitial pressure, thereby restricting substance transport. Matrix metalloproteinases (MMPs), secreted by CAFs and TAMs, degrade the ECM while recruiting MDSCs and activating CAFs^[91]^. Discoidin domain receptors (DDRs), which are transmembrane tyrosine kinase receptors, enhance collagen remodeling and thereby promote immune exclusion^[92]^. CD44, the primary receptor for hyaluronic acid (HA), promotes macrophage M2 polarization and Treg recruitment upon HA binding^[93]^. Disrupting CD44-HA interactions to target TAMs may improve immunotherapy efficacy. Although breakthroughs have been made in developing drugs targeting pathways involving LOX, MMPs, DDRs, and CD44, multi-targeted therapy is a more rational option because of the complexity of ECM-TME interactions. Research on pancreatic cancer has shown that the combined targeting of intracellular and ECM components enhances the efficacy of anti-PD-1/PD-L1 therapy^[94]^. Furthermore, Ishihara *et al.* demonstrated that conjugating anti-PD-L1 antibodies with ECM super-affinity peptides increases drug accumulation in tumor tissues while decreasing systemic concentrations, thereby enhancing efficacy and reducing adverse reactions^[95]^. Photodynamic therapy using PD-L1 immune checkpoint-targeted photoactivable liposomes (iTPALs) has also been shown to block PD-1/PD-L1 more effectively than free anti-PD-1/PD-L1 antibodies by reducing collagen density^[96]^. These preclinical findings highlight the potential of targeting the ECM to improve the efficacy of PD-1/PD-L1 inhibitors and support the development of related combination strategies for future clinical trials.

#### Targeting immune cell function

The functional state and interactions of immune cells in the TME are critical factors influencing antitumor immunity. Studies have confirmed that increasing the effector T cell/Treg ratio by targeting CD25^+^ Treg cells enhances the efficacy of anti-PD-1/PD-L1 therapy^[97]^. Another study found that Src family kinase inhibitors improve the efficacy of PD-1/PD-L1 blockade by inhibiting Treg conversion and proliferation^[98]^. Single-cell sequencing has identified neutrophil subsets associated with PD-1 inhibitor resistance, which suppress antitumor immunity by inducing irreversible T cell exhaustion^[99]^. Macrophages are another important component of the TME and are categorized as classically activated M1 and alternatively activated M2 phenotypes. M1 macrophages primarily exert proinflammatory and antitumor effects, whereas M2 macrophages exhibit immunosuppressive and protumor functions. However, macrophages in the TME are predominantly M2-like TAMs, which promote Treg differentiation and suppress effector T cell function^[100]^. TAM enrichment has been associated with resistance to ICIs, regardless of PD-L1 expression, suggesting that modulating TAMs could enhance immunotherapy^[101]^. Notably, multi-omics analyses have revealed elevated levels of M2 macrophages in patients who do not respond to anti-PD-1/PD-L1 combined with anti-angiogenic therapy^[102]^. Selective inhibition of molecules associated with M2 macrophage polarization can reprogram macrophages and overcome PD-L1 inhibitor resistance. Additionally, direct intratumoral delivery of mRNA encoding a membrane-anchored anti-CD3 single-chain variable fragment (scFv) can reprogram TAMs and promote activation of tumor-infiltrating lymphocytes. In tumor models, silencing triggering receptor expressed on myeloid cells 1 (TREM1) significantly prolonged survival. Further mechanistic research demonstrated that TREM1 deficiency enhances anti-PD-1 activity by limiting MDSC function and preventing T cell exhaustion^[103]^. Prasad *et al.* reported that early treatment with trametinib reduces MDSC abundance by suppressing CSF-1 expression, thereby sensitizing tumors to anti-PD-1 therapy, but long-term treatment restores CSF-1 expression and abolishes anti-PD-1 activity^[104]^. However, a phase I trial combining the CSF-1R inhibitor pexidartinib with durvalumab for advanced cancers showed limited clinical activity. Further analysis suggested that this may be related to the adverse effects of pexidartinib on DC differentiation through FLT3 inhibition^[105]^. Collectively, these findings suggest that targeting a single immune cell subset is inadequate for bypassing anti-PD-1/PD-L1 resistance, highlighting the necessity of multifaceted interventions to reverse the immunosuppressive TME.

### Regulating metabolism in the TME

Abnormal metabolism contributes to an immunosuppressive TME. Enhancing the therapeutic efficacy of PD-1/PD-L1 blockade through metabolic reprogramming is an emerging and promising approach. In a preclinical study of colorectal cancer, H_2_S promoted Treg activation and inhibited CD8^+^ T cell migration via ENO1 and ELK4 post-sulfation. Conversely, reducing H_2_S can enhance the antitumor activity of ICIs^[106]^. Boelaars *et al.* reported that sialic acid deficiency increases CD4^+^ and CD8^+^ T cells while reducing CD4^+^ Tregs, thereby sensitizing pancreatic cancer cells to immunotherapy^[107]^. Elevated blood ammonia levels in colorectal cancer patients were associated with non-response to anti-PD-L1 therapy^[108]^. Another study indicated that supplying L-arginine to immune cells while limiting its uptake by tumor cells may be an optimal strategy^[109]^. Dynamic labeling of L-arginine using nanomaterials can improve its delivery and boost ICI efficacy^[110]^. Other research also showed that inhibiting glutamine metabolism in tumor cells enhances immune cell function and synergizes with PD-L1 blockade^[111]^. Hypoxia in the TME is another factor contributing to anti-PD-1/PD-L1 resistance. Alleviating hypoxia through direct or indirect oxygen delivery has significantly improved antitumor efficacy. Researchers developed alginate microcapsules encapsulating cyanobacteria that generate sustained oxygen via photosynthesis. In a breast cancer mouse model, this approach demonstrated strong synergy with anti-PD-1 therapy^[112]^. A recent study proposed that targeting lipid rafts and mitochondrial respiration with albumin-bound statins can reduce hypoxia and improve PD-1 blockade efficacy in NSCLC^[113]^.

### Promoting T cell recognition and killing of tumor cells

Activated T cells recognize tumor cells through T cell receptor (TCR)-MHC-peptide interactions and eliminate them with the support of co-stimulatory molecules. Studies have shown that increasing MHC levels through PCSK9 inhibition, α-ketoglutarate supplementation, or G3BP1 inhibition can enhance T cell recognition of tumor cells^[114-116]^. Recent research revealed that valosin-containing protein (VCP) stabilizes GPD1L, causing downstream glycerol-3-phosphate (G3P) accumulation and subsequent TCR impairment. Dual inhibition of VCP and PD-1 enhanced antitumor activity in a mouse model of HCC^[117]^. The integration of PD-1/PD-L1 blockade and adoptive cell therapy (ACT) has also been actively investigated. Physical and immunologic barriers suppress ACT efficacy within solid malignancies, and the addition of PD-1/PD-L1 inhibitors could help overcome these challenges^[118]^. Giuffrida *et al.* reported that interleukin (IL)-7/IL-15 pretreatment improves chimeric antigen receptor T cell (CAR-T) responses to anti-PD-1/PD-L1 therapy^[119]^. Advances in the *ex vivo* expansion and delivery of specific effector T cells may further strengthen this combination therapy. Simultaneous blockade of PD-1 and other immune checkpoints has shown favorable safety profiles and antitumor activity in multiple phase III trials. However, limited efficacy and safety concerns in certain cancers persist, highlighting the need for further mechanistic studies^[120]^. A preclinical study suggested that a TIGIT and 4-1BB bispecific antibody (ABL112), when combined with pembrolizumab, inhibits tumor growth more effectively than pembrolizumab plus an anti-TIGIT antibody^[121]^. Hu *et al.* found that immunoradiotherapy with dual LAG-3 and TIGIT blockade effectively overcomes anti-PD-1 resistance^[122]^. Targeting multiple immune checkpoints remains a promising strategy to strengthen PD-1/PD-L1 inhibitors, and advances in multi-target inhibitors and delivery technologies may further improve clinical responses.

### Modulating host factors

Beyond the microenvironment, host factors such as age, sex, microbiota, and diet also influence the therapeutic outcomes of PD-1/PD-L1 blockade. With increasing age, lymphoid tissues undergo structural and cellular alterations that lead to diminished immune surveillance and a peripheral pro-inflammatory immune environment. This decline in immune adaptability is not only associated with increased cancer risk but also impacts the efficacy of immunotherapy^[123]^. Although immune dysfunction worsens with age across most tumor types, the roles of specific immune cell subsets are cancer type-specific. Furthermore, aging also reshapes the immune microenvironment through alterations in the ECM, blood vessels, adipocytes, and other components.

A meta-analysis showed that males may benefit more from immunotherapy, but another meta-analysis in NSCLC reported the opposite finding. Sex-related differences arise from complex interactions among genetic, hormonal, behavioral, and environmental factors. Among these, sex hormones - particularly estrogen and androgen - are key regulators of antitumor immunity and responses to ICIs. Estrogen and its receptors (ERα and ERβ) influence the formation of the TME through both tumor cell-intrinsic and -extrinsic mechanisms. Studies in NSCLC have shown that tumor cells produce estrogen through aromatase expression and activate ERα via an autocrine mechanism, thereby promoting CD274/PD-L1 overexpression^[124]^. Another preclinical study indicated that estrogen promotes the recruitment of TAMs and induces their polarization toward the M2 phenotype^[125]^. Adurthi *et al.* found that ERα signaling stimulates Treg infiltration by directly binding to the FOXP3 promoter and upregulating the expression of immunosuppressive molecules^[126]^. Furthermore, in melanoma, lung cancer, and ovarian cancer models, estrogen was shown to promote the immunosuppressive activity of MDSCs and their infiltration into tumor sites by activating the JAK2-STAT3 or TNFα pathways^[127,128]^. Preclinical models have demonstrated that blocking estrogen signaling with selective ER-degrading agents effectively restores CD8^+^ T cell function and enhances the efficacy of ICIs^[125,129]^. Notably, estrogen-mediated regulation of T cell function exhibits complexity that is dependent on receptor subtype. Binding to ERα restricts the proliferation and intratumoral infiltration of cytotoxic T cells by inducing mitochondrial dysfunction, shortening telomere length, and suppressing hTERT expression^[130,131]^. However, activation of ERβ in CD8^+^ T cells enhances TCR signaling by modulating tumor-derived phosphotyrosine switches, thereby increasing anti-PD-1 activity^[132]^. The effects of androgens in the TME primarily involve driving CD8^+^ T cell exhaustion and suppressing innate immune cell function. The androgen receptor (AR) directly impairs CD8^+^ T cell function by binding to androgen response elements (AREs) within open chromatin regions of the IFN-γ and GZMB genes, thereby suppressing their expression^[133]^. Studies in bladder cancer models have also found that AR promotes the early differentiation of CD8^+^ T cells into a “progenitor-exhausted” phenotype by upregulating the transcription factors TCF7/TCF1^[134]^. Furthermore, Liu *et al.* found that anti-androgen therapy or AR knockdown can reduce PD-L1 expression in NK cells by downregulating circ_0001005^[135]^. Based on these mechanisms, blocking androgen signaling via androgen deprivation therapy (ADT) or AR antagonists has been shown to exert synergistic effects with anti-PD-1/PD-L1 therapy in several preclinical models^[133,136]^. In addition to estrogen and androgen, progesterone and human chorionic gonadotropin (hCG) have also been found to influence T cell epigenetic and metabolic reprogramming by upregulating progesterone-induced inhibitory factors and promoting histone methylation^[9]^. Recent studies have further revealed sex-specific differences in the function of certain enzymes within T cells. In female mice, the absence of diacylglycerol acyltransferase 1 (DGAT1) in T cells enhances antitumor immunity by improving mitochondrial function and expanding precursor-depleted T cells. However, in male mice, DGAT1 deficiency leads to fatty acid peroxidation, endoplasmic reticulum stress, and T cell death via AR signaling, thereby impairing antitumor immunity^[137]^. The mechanisms underlying sex-related differences in tumor immunity and immunotherapy responses are complex. Current studies suggest that modulating sex hormones to enhance sensitivity to anti-PD-1/PD-L1 therapy is a promising direction, but further mechanistic studies are needed.

Long-distance regulation by gut microbiota and local remodeling via intratumoral microbiota together influence the TME. Gut microbiota not only contribute to local tumorigenesis but also influence tumor immunity through long-distance regulation of the TME^[138]^. Studies in mice have revealed differential effects of the gut microbiota on patient responses to anti-PD-1/PD-L1 treatment^[139]^. Fidelle *et al.* found that antibiotic-induced dysbiosis increases intestinal *Enterocloster* abundance and causes a loss of MAdCAM-1 expression, thereby triggering immune-suppressive Treg17 cell infiltration into the TME^[140]^. Another study showed that specific gut microbiota can downregulate PD-L2 and its ligand RGMb, and that blocking this pathway may overcome anti-PD-1 resistance^[141]^. Ex-Th17 cells induced by *Enterobacteriaceae* were found to produce high levels of IFN-γ and TNF in the TME, thereby promoting antitumor immunity and achieving anti-PD-1-mediated tumor control^[142]^. Furthermore, soluble metabolites from gut bacteria, such as short-chain fatty acids, inosine, and bile acids, can reach the TME via systemic circulation and influence tumor immunity^[138]^. Additional clinical evidence suggests that the use of broad-spectrum antibiotics disrupts the gut microbiome, thereby impairing immunotherapy efficacy^[143]^. Conversely, increased gut microbiota diversity (e.g., *Bifidobacterium spp*.) has been associated with higher response rates to ICIs^[139]^. Modulation of the gut microbiota via fecal microbiota transplantation has also been shown to enhance the efficacy of PD-1 inhibitors in early clinical trials^[144]^. Recently, a synthetic microbiota composed of 15 gut bacterial strains was successfully engineered and demonstrated the potential to reverse anti-PD-1 antibody resistance, regardless of individual gut microbiota backgrounds^[145]^. Unlike gut microbiota, microorganisms colonizing tumor sites directly participate in TME remodeling. Although the biomass of intratumoral bacteria is low (typically 1-10 bacteria per 100,000 tumor cells)^[146]^, these microorganisms can influence the TME through direct contact and local metabolism. *H. pylori* and *S. Typhimurium* promote immune evasion by inducing PD-L1 expression in host cells^[147,148]^. Hezaveh *et al.* found that indole metabolites secreted by *Lactobacillus* promote the polarization of TAMs toward an immunosuppressive phenotype by activating the AhR pathway^[149]^. Conversely, some bacteria are considered beneficial for immunotherapy, with baseline enrichment of *Clostridium* at tumor sites found to be associated with response to ICIs^[150]^. Intratumoral injection of *Burkholderia cepacia*, *Priestia megaterium*, or *Corynebacterium kroppenstedtii* in melanoma-bearing mice was found to synergistically inhibit tumor growth when combined with anti-PD-1 therapy^[151]^. With the deepening understanding of the microbiome, clinical intervention strategies are shifting toward precision medicine. Canale *et al.* found that engineered tumor-colonizing *Escherichia coli* can convert ammonia into L-arginine, thereby improving immunotherapy efficacy^[152]^.

In addition to the factors discussed above, diet, exercise, and chronic stress may also influence tumor immunity and responses to anti-PD-1/PD-L1 therapy. Preclinical studies have shown that a ketogenic diet alters the immunosuppressive TME through multiple mechanisms, increasing sensitivity to ICIs^[153]^. Regular exercise has been shown to enhance NK cell cytotoxicity and reshape the T cell repertoire, supporting antitumor immunity^[154]^. Growing interest in host-related factors underscores the potential of modulating host physiology to improve immunotherapy efficacy.

### Targeting neuro-immune crosstalk

With increasing research attention focused on the neuro-immune system, its role in ICI resistance has gradually been recognized in recent years. Abnormal nerve fiber growth or irregular neurotransmitter secretion can lead to immune escape. Targeting neurotransmitters and their receptors has shown potential for overcoming resistance. Plasma neurotransmitter analyses in patients who were sensitive or resistant to anti-PD-1 antibodies revealed that norepinephrine influences CXCL9 and adenosine (ADO) secretion in tumor cells, thereby inhibiting the chemotaxis and function of CD8^+^ T cells^[155]^. Qiao *et al.* reported that norepinephrine inhibits TCR signaling by activating β-adrenergic receptors (β-ARs) on CD8^+^ T cells while promoting metabolic dysfunction and exhaustion^[156]^. A meta-analysis showed that β-blocker use may correlate with improved ICI efficacy^[157]^. The safety and antitumor activity of combining β-AR antagonists with PD-1/PD-L1 inhibitors are being investigated in several clinical trials. In contrast to the β-AR pathway, α2-adrenergic receptor (α2-AR) agonists have shown the ability to induce MDSC apoptosis and enhance CD8^+^ T cell infiltration, thereby exhibiting effective antitumor activity even in ICI-resistant tumor models^[158]^. Furthermore, activation of the α7 nicotinic acetylcholine receptor has been shown to suppress TNF-α release in macrophages and modulate T cell function^[159]^. Schneider *et al.* revealed that platelet-derived serotonin upregulates PD-L1 on cancer cells, thereby driving CD8^+^ T cell exhaustion^[160]^. Combining serotonin-targeting drugs with PD-1/PD-L1 inhibitors has achieved long-term tumor inhibition. Further studies have also revealed the role of sensory neurons in tumor immunity. In preclinical melanoma models, calcitonin gene-related peptide (CGRP), released by nociceptors, increased CD8^+^ T cell exhaustion^[161]^. Although the mechanism by which CGRP regulates T cells remains incompletely understood, it may involve Gαs signaling mediated by the RAMP1–CALCRL receptor complex^[162]^. In summary, neurotransmitter signaling pathways play critical roles in resistance to PD-1/PD-L1 inhibitors. Further studies in this field may reveal novel therapeutic targets and combination strategies for overcoming immunotherapy resistance.

## CONCLUSION AND FUTURE PERSPECTIVES

Recent years have seen PD-1/PD-L1 inhibitors revolutionize the landscape of antitumor therapy, but primary and acquired resistance remain substantial clinical challenges. Based on analyses of clinical and preclinical evidence, this review supports the view that mechanism-driven combination therapy is an inevitable trend toward achieving durable and effective immunotherapy responses. The success of OPDUALAG and ivonescimab has highlighted the clinical value of dual-target regimens, further driving the development of dual- or multi-target drugs. However, some combination regimens, such as anti-PD-L1 plus anti-TGF-β, have shown inconsistent efficacy or additive toxicity in clinical trials. These findings suggest that not all combinations are viable, and that combination strategies should be based on interpretable synergistic efficacy and manageable safety. One direction for future research is the development of conditionally active antibodies, ADCs, and nanomedicine-based delivery systems that restrict immune activation to the TME, thereby maximizing efficacy while minimizing systemic toxicity.

Furthermore, the mechanisms of resistance to PD-1/PD-L1 inhibitors exhibit high heterogeneity, suggesting that precision-tailored treatment regimens for tumors with different immune phenotypes may more effectively overcome resistance. For tumors characterized by dense stroma and immune exclusion, such as pancreatic cancer, improving immune cell infiltration by targeting the ECM and CAFs may improve the efficacy of anti-PD-1/PD-L1 therapy. Conversely, for tumors characterized by immune cell exhaustion, restoring T cell cytotoxicity through pathways such as metabolic reprogramming holds greater potential for achieving synergistic effects.

Notably, the influence of host factors on immunotherapy should be considered in future preclinical and clinical studies. Early trials have already recognized the value of modulating the gut microbiota and implementing sex hormone-based interventions to improve immunotherapy outcomes, marking a transition within the therapeutic paradigm from a tumor-centric to a host-centric approach. The next breakthrough in cancer immunotherapy will rely on dynamic, multi-omics-guided analyses to design personalized, multi-target treatment regimens. By comprehensively considering the intrinsic mechanisms of tumors, the TME, and host factors, the ultimate goal is to convert non-responders into responders, thereby achieving durable treatment responses in a broader patient population.

## DECLARATIONS

### Copyright

© The Author(s) 2026.
