# Supplementary material for: Overcoming resistance to anti-PD-1/PD-L1 therapy in cancer
Source: Cancer Drug Resist. 2026 May 15;9:18. doi: 10.20517/cdr.2026.12 (PMC13244269; doi:10.20517/cdr.2026.12)
Supplement: Supplementary file 1 [file cdr-9-18-SupplementaryMaterials.pdf]

Supplementary Materials

Overcoming resistance to anti-PD-1/PD-L1 therapy in cancer

Lijun Li[1,2,3,#], Yanbin Zhao[3,#], Xinhong Shi[1,2], Xiaotian Guo[3], Xiaoxin Zhang[1,2], Guangrui Li[3], Qingwei Meng[3], Minghui Zhang[1,2], Mingzhu Yin[2,4]

[1]Clinical Research Center (CRC), Chongqing University Three Gorges Hospital, Chongqing University, Chongqing 404100, China.
[2]School of Medicine Chongqing University, Chongqing University, Chongqing 400030, China.
[3]Department of Medical Oncology, Harbin Medical University Cancer Hospital, Harbin 150081, Heilongjiang, China.
[4]Clinical Research Center (CRC), Medical Pathology Center (MPC), Cancer Early Detection and Treatment Center (CEDTC) and Translational Medicine Research Center (TMRC), Chongqing University Three Gorges Hospital, Chongqing University, Chongqing 404100, China.
[#]Authors contributed equally.

Correspondence to: Prof. Qingwei Meng, Department of Medical Oncology, Harbin Medical University Cancer Hospital, Harbin 150081, Heilongjiang, China. E-mail: mengqw@hrbmu.edu.cn; Prof. Minghui Zhang, Prof. Mingzhu Yin, School of Medicine Chongqing University, Chongqing University, Chongqing 400030, China. E-mail: zhmhui@cqu.edu.cn; yinmingzhu@cqu.edu.cn

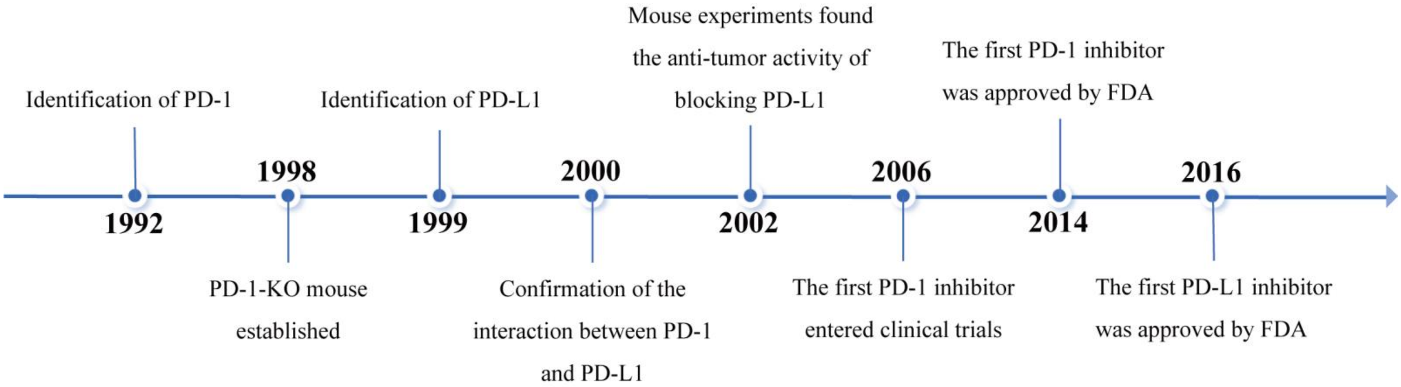

Supplementary Figure 1. Timeline for major events leading to the development of anti-PD-1/PD-L1 drugs.

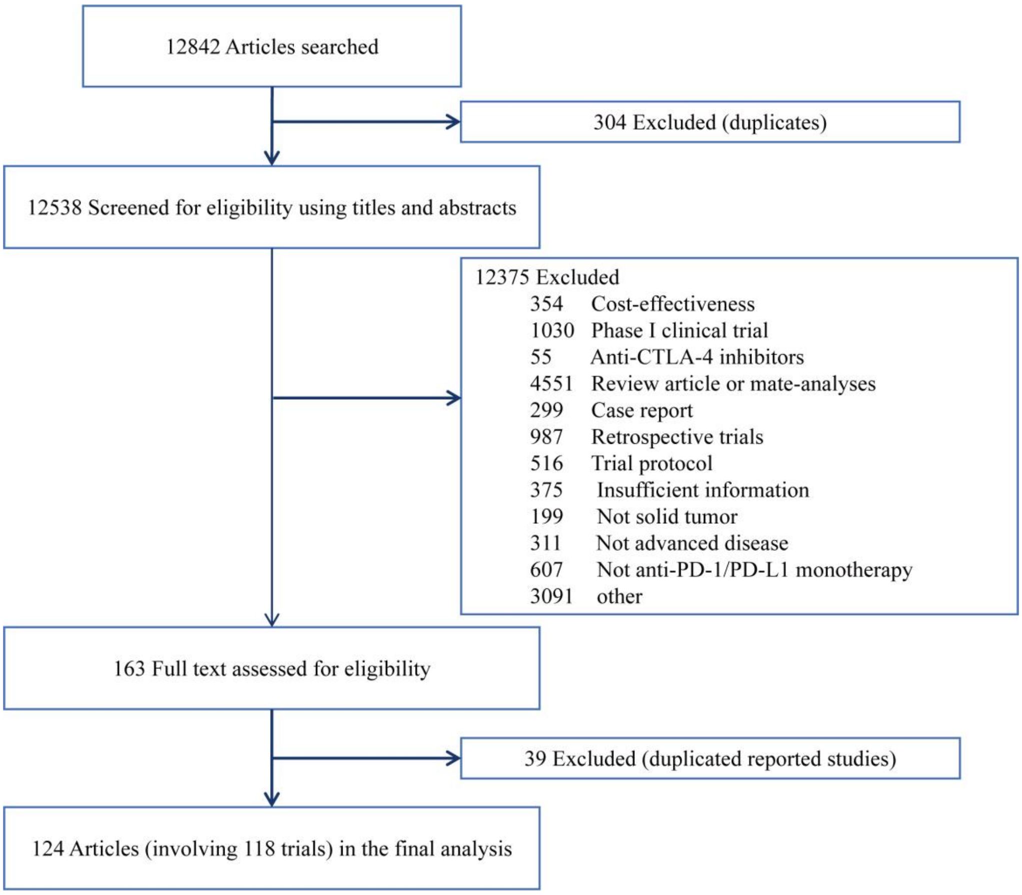

Supplementary Figure 2. Flow diagram for the selection of eligible studies to evaluation the objective response rate of anti-PD-1/PD-L1 monotherapy.

Supplementary Table 1. Search strategy for PD-1/PD-L1 inhibitor related clinical trials in PubMed

PATIENT	#1	((cancer[Title/Abstract]) OR (carcinoma[Title/Abstract])) OR (neoplasm)
INTERVENTION	#2	(((((((((((((((((((((((((((((((((((((Immune Checkpoint Inhibitor[Title/Abstract]) OR (ICI[Title/Abstract])) OR (immune therapy[Title/Abstract])) OR (immunotherapy[Title/Abstract])) OR (programmed cell death protein-1[Title/Abstract])) OR (programmed cell death protein[Title/Abstract])) OR (PD-1[Title/Abstract])) OR (nivolumab[Title/Abstract])) OR (pembrolizumab[Title/Abstract])) OR (camrelizumab[Title/Abstract])) OR (sintilimab[Title/Abstract])) OR (toripalimab[Title/Abstract])) OR (tislelizumab[Title/Abstract])) OR (iparomlimab[Title/Abstract])) OR (enlonstobart[Title/Abstract])) OR (ivonescimab[Title/Abstract])) OR (retifanlimab[Title/Abstract])) OR (pucotenlimab[Title/Abstract])) OR (cadonilimab[Title/Abstract])) OR (serplulimab[Title/Abstract])) OR (zimberelimab[Title/Abstract])) OR (penpulimab[Title/Abstract])) OR (dostarlimab[Title/Abstract])) OR (prolgolimab[Title/Abstract])) OR (finotonlimab[Title/Abstract])) OR (programmed cell death-Ligand 1[Title/Abstract])) OR (PD-L1[Title/Abstract])) OR (atezolizumab[Title/Abstract])) OR (durvalumab[Title/Abstract])) OR (avelumab[Title/Abstract])) OR (cemiplimab[Title/Abstract])) OR (KL-A167[Title/Abstract])) OR (benmelstobart[Title/Abstract])) OR (socazolimab[Title/Abstract])) OR (adebrelimab[Title/Abstract])) OR (cosibelimab[Title/Abstract])) OR (sugemalimab[Title/Abstract])) OR (envafolimab[Title/Abstract])
STUDY TYPE	#3	(((((((((((((((((((((((((((((((((randomized controlled trial[Title/Abstract]) OR (controlled clinical trial[Title/Abstract])) OR (randomized[Title/Abstract])) OR (placebo[Title/Abstract])) OR (randomly[Title/Abstract])) OR (trial[Title/Abstract])) OR (RCT[Title/Abstract])) OR (phase III[Title/Abstract])) OR (phase 3 clinical trial[Title/Abstract])) OR (phase III clinical trial[Title/Abstract])) OR (phase 3 trial[Title/Abstract])) OR (phase III trial[Title/Abstract])) OR (phase 3 clinical study[Title/Abstract])) OR (phase III clinical study[Title/Abstract])) OR (phase 3 study[Title/Abstract])) OR (phase III study[Title/Abstract])) OR (phase 3 randomized trial[Title/Abstract])) OR (phase III randomized trial[Title/Abstract])) OR (Phase II[Title/Abstract])) OR (phase 2 clinical trial[Title/Abstract])) OR （phase II clinical trial[Title/Abstract])) OR (phase 2 trial[Title/Abstract])) OR (phase II trial[Title/Abstract])) OR (phase 2 clinical study[Title/Abstract])) OR (phase II clinical study[Title/Abstract])) OR (phase 2 randomized trial[Title/Abstract])) OR (phase II randomized trial[Title/Abstract])) OR (phase 2 study[Title/Abstract])) OR (phase II study[Title/Abstract])) OR (phase 2/3 clinical trial[Title/Abstract])) OR (phase II/III clinical trial[Title/Abstract])) OR (phase 2/3 trial[Title/Abstract])) OR (phase II/III trial[Title/Abstract])) OR (phase 2/3 clinical study[Title/Abstract])) OR (phase II/ III clinical study[Title/Abstract])) OR (phase 2/3 study[Title/Abstract])) OR (phase II/III study[Title/Abstract])) OR (phase 2/3 randomized trial[Title/Abstract])) OR (phase II/III randomized trial[Title/Abstract])
SEARCH STRATEGY	#1 AND #2 AND #3 s: from 2010/1/1 - 2025/4/30	

Supplementary Table 2. The clinical trials of PD-1/PD-L1 inhibitors as monotherapy in advanced cancers

Study	Trial ID	Phase	Cancer type	Line of therapy	Treatment	Number of patients	ORR (95% CI)	Ref.
CheckMate-143	NCT02017717	III	Glioblastoma	2L	Nivolumab	153	7.8% (4.1%-13.3%)	[1]
KEYNOTE-048	NCT02358031	III	Head and neck cancer	1L	Pembrolizumab	301	17%	[2]
CheckMate-714	NCT02823574	II	Head and neck cancer (platinum-refractory)	1L	Nivolumab	82	19.5% (11.6%-29.7%)	[3]
CheckMate-714	NCT02823574	II	Head and neck cancer (platinum-eligible)	1L	Nivolumab	61	29.5% (18.5%-42.6%)	[3]
EAGLE	NCT02369874	III	Head and neck cancer	2L	Durvalumab	240	17.9% (13.3%–23.4%)	[4]
Checkmate-141	NCT02105636	III	Head and neck cancer	≥2L	Nivolumab	240	13.3% (9.3%-18.3%)	[5]
KEYNOTE-040	NCT02252042	III	Head and neck cancer	≥2L	Pembrolizumab	247	14.6% (10.4%–19.6%)	[6]
CONDOR	NCT02319044	II	Head and neck cancer	2L	Durvalumab	65	9.2% (3.46%-19.02%)	[7]
HAWK	NCT02207530	II	Head and neck cancer	2L	Durvalumab	111	16.2% (9.9%-24.4%)	[8]
KEYNOTE-122	NCT02611960	III	Nasopharyngeal cancer	2L	Pembrolizumab	117	21.4% (14.3%-29.9%)	[9]
CPDR001X2201	NCT03866967	II	Nasopharyngeal cancer	2L	Spartalizumab	82	17.1% (9.7% to 27.0%)	[10]
JS001-Ib-CRP-1.0	NCT02915432	II	Nasopharyngeal cancer	≥3L	Penpulimab	125	28.0% (20.3%–36.7%)	[11]
POLARIS-02	NCT02605967	II	Nasopharyngeal cancer	≥3L	Toripalimab	190	20.5% (15.0%-27.0%)	[12]
FLAT	NCT05783882	III	Melanoma	1L	Prolgolimab (250mg)	114	33.3% (24.8%-42.8%）	[13]
FLAT	NCT05783882	III	Melanoma	1L	Prolgolimab (1mg/kg)	61	32.8% (21.3%-46.0%)	[13]
/	JapicCTI-142533	II	Melanoma	1L	Nivolumab	23	34.8% (20.8%-51.9%)	[14]
CARSKIN	NCT02883556	II	Cutaneous squamous cell carcinoma	1L	Pembrolizumab	57	42% (29%-56%)	[15]
NIVOSQUACS	NCT04204837	II	Cutaneous squamous cell carcinoma	1L	Nivolumab	31	61.3% (42.2%–78.2%)	[16]
R2810-ONC-1620	NCT03132636	II	Basal cell carcinoma	2L	Cemiplimab	84	31% (21%-42%)	[17]
KEYNOTE-913	NCT03783078	III	Merkel cell carcinoma	1L	Pembrolizumab	55	49% (35%–63%)	[18]
KEYNOTE-017	NCT02267603	II	Merkel cell carcinoma	1L	Pembrolizumab	50	58% (43.2%-71.8%)	[19]
JAVELIN Merkel-200	NCT02155647	II	Merkel cell carcinoma	≥2L	Avelumab	88	33.0% (23.3%-43.8%)	[20]
KEYNOTE-062	NCT02494583	III	Gastric cancer	1L	Pembrolizumab	256	14.8%	[21]
ONO-4538-12	NCT02267343	III	Gastric or	≥3L	Nivolumab	330	11.2%	[22]

			gastro-oesophageal junction cancer				(7.7%–15.6%)	
JAVELIN Gastric-300	NCT02625623	III	Gastric or gastro-oesophageal junction cancer	3L	Avelumab	185	2.2% (0.6%-5.4%)	[23]
KEYNOTE-061	NCT02370498	III	Gastric or gastro-oesophageal junction cancer	≥2L	Pembrolizumab	196	16% (11%-22%)	[24]
GS-US-296-2013	NCT02864381	II	Gastric cancer	≥2L	Nivolumab	72	6.9% (2.3%-15.5%)	[25]
MCC-18684	NCT02829918	II	Biliary tract cancer	≥2L	Nivolumab	46	22%	[26]
CheckMate-459	NCT02576509	III	Hepatocellular carcinoma	1L	Nivolumab	371	15% (12%–19%)	[27]
RATIONALE-301	NCT03412773	III	Hepatocellular carcinoma	1L	Tislelizumab	342	14.3% (10.8%-18.5%)	[28]
KEYNOTE-224 (cohort 2)	NCT02702414	II	Hepatocellular carcinoma	1L	Pembrolizumab	51	16% (7%–29%)	[29]
KEYNOTE-240	NCT02702401	III	Hepatocellular carcinoma	2L	Pembrolizumab	278	18.3% (14.0%-23.4%）	[30]
KEYNOTE-394	NCT03062358	III	Hepatocellular carcinoma	2L	Pembrolizumab	300	12.7% (9.1%-17.0%)	[31]
SHR-1210-II/III-HCC	NCT02989922	II	Hepatocellular carcinoma	≥2L	Camrelizumab	217	14.7% (10.3%–20.2%)	[32]
KEYNOTE-224(cohort 1)	NCT02702414	II	Hepatocellular carcinoma	2L	Pembrolizumab	104	18.3% (11.4%-27.1%)	[33]
RATIONALE-208	NCT03419897	II	Hepatocellular carcinoma	≥2L	Tislelizumab	249	13% (9%-18%)	[34]
KEYNOTE-177	NCT02563002	III	MSI-H/dMMR colorectal cancer	1L	Pembrolizumab	153	45.8% (37.7%-54.0%)	[35]
CheckMate-142	NCT02060188	II	MSI-H/dMMR colorectal cancer	≥2L	Nivolumab	74	32.4% (22.0%-44.3%)	[36]
/	NCT03435107	II	MSI-H/dMMR or POLE-mutated colorectal cancer	≥2L	Durvalumab	33	42.4% (25.5%-60.8%)	[37]
KEYNOTE-164 (cohort A)	NCT02460198	II	MSI-H/dMMR colorectal cancer	≥3L	Pembrolizumab	61	32.8% (21.3%–46.0%)	[38]
KEYNOTE-164 (cohort B)	NCT02460198	II	MSI-H/dMMR colorectal cancer	≥2L	Pembrolizumab	63	34.9% (23.3%–48.0%)	[38]
KEYNOTE-158	NCT02628067	II	Anal squamous cell carcinoma	≥2L	Pembrolizumab	112	11% (6%-18%)	[39]
POD1UM-202	NCT03597295	II	Anal squamous cell carcinoma	≥2L	Retifanlimab	94	13.8% (7.6%-22.5%)	[40]
NCI9673	NCT02314169	II	Anal cancer, except adenocarcinoma	≥2L	Nivolumab	37	24% (15%–33%)	[41]
KEYNOTE-181	NCT02564263	III	Esophageal cancer	2L	Pembrolizumab	314	13.1% (9.5%-17.3%)	[42]
RATIONALE-302	NCT03430843	III	Esophageal squamous cell carcinoma	2L	Tislelizumab	256	20.3% (15.6%-25.8%)	[43]
ESCORT	NCT03099382	III	Esophageal squamous	2L	Camrelizumab	228	20.2%	[44]

			cell carcinoma				(15.2%–26.0%)	
ATTRACTION-3	NCT02569242	III	Esophageal squamous cell carcinoma	2L	Nivolumab	210	19% (14%–26%)	[45]
CheckMate-032	NCT01928394	III	Esophageal cancer	≥2L	Nivolumab	59	12% (5%-23%)	[46]
KEYNOTE-180	NCT02559687	II	Esophageal cancer	≥3L	Pembrolizumab	121	9.9% (5.2%-16.7%)	[47]
ORIENT-2	NCT03116152	II	Esophageal squamous cell carcinoma	2L	Sintilimab	95	12.6% (6.7%–21.0%)	[48]
/	NCT02971956	II	Esophageal cancer	≥2L	Pembrolizumab	49	8% (2.3%-19.6%)	[49]
/	JapicCTI-No. 142422	II	Esophageal squamous cell carcinoma	≥2L	Nivolumab	64	17% (10%–28%)	[50]
DIADEM	NCT04115111	II	Malignant pleural mesothelioma	2L	Durvalumab	58	10.3% (3.9%-21.2%)	[51]
MERIT	JapicCTI163247	II	Malignant pleural mesothelioma	2-3L	Nivolumab	34	29.4% (16.8%–46.2%)	[52]
/	NCT02364076	II	Thymic carcinoma	≥2L	Pembrolizumab	40	22.5% (10.8%–38.5%)	[53]
/	NCT02607631	II	Thymic carcinoma	≥2L	Pembrolizumab	26	19.2% (8.5%-37.9%)	[54]
ML41253	NCT04321330	II	Thymic carcinoma	≥2L	Atezolizumab	34	14.7% (5.0%-31.1%)	[55]
PRIMER	UMIN000022007	II	Thymic carcinoma	≥2L	Nivolumab	15	0% (0%-21.8%)	[56]
IFCT-1603	NCT03059667	II	Small cell lung cancer	2L	Atezolizumab	43	2.3% (0–6.8%)	[57]
CheckMate-026	NCT02041533	III	Non-small cell lung cancer	1L	Nivolumab	211	26% (20%–33%)	[58]
KEYNOTE-024	NCT02142738	III	Non-small cell lung cancer	1L	Pembrolizumab	154	44.8% (36.8%-53.0%)	[59]
EMPOWER-Lung 1	NCT03088540	III	Non-small cell lung cancer	1L	Cemiplimab	283	39% (34%–45%)	[60]
KEYNOTE-042	NCT02220894	III	Non-small cell lung cancer	1L	Pembrolizumab	637	27.3% (23.9%-31.0%)	[61]
IPSOS	NCT03191786	III	Non-small cell lung cancer	1L	Atezolizumab	302	17% (12.8%-21.6%)	[62]
JAVELIN Lung 100	NCT02576574	III	Non-small cell lung cancer	1L	Avelumab (Q2W)	366	25.4% (21.0%-30.2%)	[63]
JAVELIN Lung 100	NCT02576574	III	Non-small cell lung cancer	1L	Avelumab (QW)	322	23.3% (18.8%-28.3%)	[63]
/	NCT02879617	II	Non-small cell lung cancer	1L	Durvalumab	38	26% (13%–43%)	[64]
KEYNOTE-654/ECHO-305	NCT03322540	II	Non-small cell lung cancer	1L	Pembrolizumab	77	39.0% (28.0%–50.8%）	[65]
POD1UM-203 (NSCLC cohort)	NCT03679767	II	Non-small cell lung cancer	1L	Retifanlimab	23	34.8% (16.4%-57.3%)	[66]
CTONG1901 (arm A)	NCT04252365	II	Non-small cell lung cancer	1L	Sintilimab	13	46.2% (19.2%-74.9%)	[67]

CTONG1901 (arm B)	NCT04252365	II	Non-small cell lung cancer	1L	Pembrolizumab	14	42.9% (17.7%-71.1%)	[67]
SAKK 19/17	NCT03620669	II	Non-small cell lung cancer	1L	Durvalumab	48	17% (8%-30%)	[68]
BIRCH (cohort 1)	NCT02031458	II	Non-small cell lung cancer	1L	Atezolizumab	139	22% (15%-29%)	[69]
OAK	NCT02008227	III	Non-small cell lung cancer	≥2L	Atezolizumab	613	13.7% (11.1%–16.7%)	[70]
TAIL	NCT03285763	III/IV	Non-small cell lung cancer	≥2L	Atezolizumab	615	11.1% (8.7%-13.8%)	[71]
CheckMate-057	NCT01673867	III	Non-small cell lung cancer	≥2L	Nivolumab	292	19% (15%-24%)	[72]
CheckMate-017	NCT01642004	III	Non-small cell lung cancer	≥2L	Nivolumab	135	20% (14%-28%)	[73]
CheckMate-870	NCT03195491	III	Non-small cell lung cancer	2L	Nivolumab	400	15% (11.65%-18.88 %)	[74]
CheckMate-078	NCT02613507	III	Non-small cell lung cancer	2L	Nivolumab	338	18% (13.6%-21.9%)	[75]
ORIENT-3	NCT03150875	III	Non-small cell lung cancer	2L	Sintilimab	145	25.50% (18.60%-33.40 %)	[76]
RATIONALE-303	NCT03358875	III	Non-small cell lung cancer	2-3L	Tislelizumab	535	22.6% (19.1%-26.4%)	[77]
KEYNOTE-033	NCT02864394	III	Non-small cell lung cancer	≥2L	Pembrolizumab	213	20.7% (15.4%-26.7%)	[78]
JAVELIN Lung 200	NCT02395172	III	Non-small cell lung cancer	2L	Avelumab	396	15.2% (11.8%–19.1%)	[79]
BIRCH (cohort 2)	NCT02031458	II	Non-small cell lung cancer	2L	Atezolizumab	268	19% (15%-25%)	[69]
BIRCH (cohort 3)	NCT02031458	II	Non-small cell lung cancer	≥3L	Atezolizumab	252	18% (13%-23%)	[69]
ONO-4538-09	NCT02175017	II	Non-small cell lung cancer	2-3L	Nivolumab	100	21.0% (14.2%-30.0%)	[80]
SWOG S1400A	NCT02154490	II/III	Non-small cell lung cancer	≥2L	Durvalumab	68	16% (7%-25%)	[81]
CheckMate-171	NCT02409368	II	Non-small cell lung cancer	≥2L	Nivolumab	472	11.0% (8.3%-14.2%)	[82]
ONO-4538-25	NCT02582125	II	Non-small cell lung cancer	≥2L	Nivolumab	53	9.4% (4.10%-20.25%)	[83]
SHR-1210-II-201-NSCLC	NCT03085069	II	Non-small cell lung cancer	≥2L	Camrelizumab	146	17.8% (12.0%-25.0%)	[84]
NIDO-101	NCT04023617	II	Non-small cell lung cancer	2L	Nivolumab	10	10% (0-28.6%)	[85]
/	JapicCTI132072	II	Non-small cell lung cancer	≥2L	Nivolumab	35	25.7% (14.2%-42.1%)	[86]
/	JapicCTI132073	II	Non-small cell lung cancer	≥2L	Nivolumab	76	22.4% (14.5%-32.9%).	[87]
WJOG9616L	UMIN000028561	II	Non-small cell lung	≥2L	Nivolumab	59	8.5%	[88]

			cancer				(2.8%-18.7%)	
KEYNOTE-427 (cohort A)	NCT02853344	II	Clear cell renal cell carcinoma	1L	Pembrolizumab	110	36.4% (27.4%-46.1%)	[89]
KEYNOTE-427 (cohort B)	NCT02853344	II	Non–clear cell renal cell carcinoma	1L	Pembrolizumab	165	26.7% (20.1%-34.1%)	[90]
HCRN GU16-260 (cohort A)	NCT03117309	II	Clear cell renal cell carcinoma	1L	Nivolumab	123	34.1% (25.8%-43.2%).	[91]
HCRN GU16-260 (cohort B)	NCT03117309	II	Non–clear cell renal cell carcinoma	1L	Nivolumab	35	14.3% (4.8%-30.3%)	[92]
POD1UM-203 (RCC cohort)	NCT03679767	II	Clear cell renal cell carcinoma	1L	Retifanlimab	34	23.5% (10.7%-41.2%)	[66]
CheckMate-025	NCT01668784	III	Renal cell carcinoma	2-3L	Nivolumab	410	22.9% (18.9%-27.3%)	[93]
CA209-010	NCT01354431	II	Renal cell carcinoma	≥2L	Nivolumab (0.3mg/kg)	60	20% (13.4%-28.2%)	[94]
CA209-010	NCT01354431	II	Renal cell carcinoma	≥2L	Nivolumab (2mg/kg)	54	22% (15.0%-31.1%)	[94]
CA209-010	NCT01354431	II	Renal cell carcinoma	≥2L	Nivolumab (10mg/kg)	54	20% (13.4%-29.1%)	[94]
/	NCT02118337	II	Clear cell renal cell carcinoma	≥2L	Nivolumab	21	23.8% (8.2%-47.2%)	[95]
/	NCT02673333	II	Adrenocortical carcinoma	/	Pembrolizumab	39	23% (11%-39%)	[96]
NU 15E01	NCT02720484	II	Adrenocortical carcinoma	≥1L	Nivolumab	10	10%	[97]
IMvigor130	NCT02807636	III	Urothelial carcinoma	1L	Atezolizumab	359	23% (19%-28%)	[98]
KEYNOTE-361	NCT02853305	III	Urothelial carcinoma	1L	Pembrolizumab	307	30.3% (25.2%-35.8%)	[99]
KEYNOTE-052	NCT02335424	II	Urothelial carcinoma	1L	Pembrolizumab	370	28.6% (24.1%-33.5%)	[100]
IMvigor210 (cohort 1)	NCT02951767	II	Urothelial carcinoma	1L	Atezolizumab	119	23.5% (16.2%-32.2%)	[101]
IMvigor210 (cohort 2)	NCT02108652	II	Urothelial carcinoma	≥2L	Atezolizumab	310	16.5% (12.5%-21.1%)	[101]
KEYNOTE-045	NCT02256436	III	Urothelial carcinoma	2L	Pembrolizumab	270	21.1% (16.4%-26.5%)	[102]
IMvigor211	NCT02302807	III	Urothelial carcinoma	≥2L	Atezolizumab	462	13.4% (10.5%-16.9%)	[103]
SAUL (IMvigor211-like)	NCT02928406	III	Urothelial carcinoma	≥2L	Atezolizumab	643	14% (11%-17%)	[104]
CheckMate-275	NCT02387996	II	Urothelial carcinoma	≥2L	Nivolumab	265	19.6% (15.0%-24.9%)	[105]
KEYNOTE-143	NCT02351739	II	Urothelial carcinoma	≥2L	Pembrolizumab	35	25.7% (12.5%-43.3%)	[106]
BGB-A317-204	NCT04004221	II	Urothelial carcinoma	≥2L	Tislelizumab	104	24% (16%-33%)	[107]
POLARIS-03	NCT03113266	II	Urothelial carcinoma	≥2L	Toripalimab	151	26%	[108]

							(19%-34%)	
KEYNOTE-158	NCT02628067	II	MSI-H/dMMR cancer	≥1L	Pembrolizumab	233	34.3% (28.3%-40.8%)	[109]
DRUP	NCT02925234	II	MSI-H/dMMR cancer	≥1L	Nivolumab	130	45% (35.9%-53.6%)	[110]
PERICLES	NCT03686332	II	Penile squamous cell carcinoma	≥1L	Atezolizumab	30	16.7% (6%-35%)	[111]
ORPHEUS	NCT04231981	II	Penile squamous cell carcinoma	≥1L	Retifanlimab	18	16.7% (5.8%-39.2%)	[112]
KEYNOTE-199	NCT02787005	II	Prostate cancer	≥2L	Pembrolizumab	199	5% (2%-8%)	[113]
PICK-NEPC	NCT03179410	II	Prostate cancer	≥2L	Avelumab	15	6.7%	[114]
KEYNOTE-086	NCT02447003	II	Breast cancer	≥2L	Pembrolizumab	170	5.3% (2.7-9.9%)	[115]
KEYNOTE-158	NCT02628067	II	MSI-H/dMMR endometrial cancer	≥2L	Pembrolizumab	79	48% (37%-60%)	[116]
KEYNOTE-158	NCT02628067	II	Cervical cancer	≥2L	Pembrolizumab	77	14.3% (7.4%-24.1%)	[117]
/	NCT03104699	II	Cervical cancer	≥2L	Balstilimab	140	15.0% (10.0%-21.8%)	[118]
EMPOWER-Cervical 1	NCT03257267	III	Cervical cancer	≥2L	Cemiplimab	304	16.4% (12.5%-21.1%)	[119]
YH-S001-05	NCT03972722	II	Cervical cancer	≥2L	Zimberelimab	105	27.6 (19.34%-37.20%)	[120]
SKYSCRAPER-04	NCT04300647	II	Cervical cancer	≥2L	Atezolizumab	45	15.6% (6.5%-29.5%)	[121]
JAVELIN Ovarian 200	NCT02580058	III	Ovarian cancer	≥2L	Avelumab	188	4% (2%-8%)	[122]
NINJA	JapicCTI153004	III	Ovarian cancer	≥2L	Nivolumab	119	7.6% (3.5%-13.9%）	[123]
KEYNOTE-100	NCT02674061	II	Ovarian cancer	≥2L	Pembrolizumab	376	8.0% (5.4%-11.2%)	[124]

REFERENCE

1. [1]. Reardon DA, Brandes AA, Omuro A et al. Effect of Nivolumab vs Bevacizumab in Patients With Recurrent Glioblastoma: The CheckMate 143 Phase 3 Randomized Clinical Trial. *JAMA Oncol* 2020, 6:1003–10. PMID:32437507 doi: 10.1001/jamaoncol.2020.1024
2. [2]. Burtness B, Harrington KJ, Greil R et al. Pembrolizumab alone or with chemotherapy versus cetuximab with chemotherapy for recurrent or metastatic squamous cell carcinoma of the head and neck (KEYNOTE-048): a randomised, open-label, phase 3 study. *Lancet* 2019, 394:1915–28. PMID:31679945 doi: 10.1016/s0140-6736(19)32591-7
3. [3]. Harrington KJ, Ferris RL, Gillison M et al. Efficacy and Safety of Nivolumab Plus Ipilimumab vs Nivolumab Alone for Treatment of Recurrent or Metastatic Squamous Cell Carcinoma of the Head and Neck: The Phase 2 CheckMate 714 Randomized Clinical Trial. *JAMA Oncol* 2023, 9:779–89. PMID:37022706 doi: 10.1001/jamaoncol.2023.0147
4. [4]. Ferris RL, Haddad R, Even C et al. Durvalumab with or without tremelimumab in patients with recurrent or metastatic head and neck squamous cell carcinoma: EAGLE, a randomized, open-label phase III study. *Ann Oncol* 2020, 31:942–50. PMID:32294530 doi: 10.1016/j.annonc.2020.04.001
5. [5]. Ferris RL, Blumenschein G, Jr., Fayette J et al. Nivolumab for Recurrent Squamous-Cell Carcinoma of the Head and Neck. *N Engl J Med* 2016, 375:1856–67. PMID:27718784 doi: 10.1056/NEJMoa1602252
6. [6]. Cohen EEW, Soulières D, Le Tourneau C et al. Pembrolizumab versus methotrexate, docetaxel, or cetuximab for recurrent or metastatic head-and-neck squamous cell carcinoma (KEYNOTE-040): a randomised, open-label, phase 3 study. *Lancet* 2019, 393:156–67. PMID:30509740 doi: 10.1016/s0140-6736(18)31999-8
7. [7]. Siu LL, Even C, Mesía R et al. Safety and Efficacy of Durvalumab With or Without Tremelimumab in Patients With PD-L1-Low/Negative Recurrent or Metastatic HNSCC: The Phase 2 CONDOR Randomized Clinical Trial. *JAMA Oncol* 2019, 5:195–203. PMID:30383184 doi: 10.1001/jamaoncol.2018.4628
8. [8]. Zandberg DP, Algazi AP, Jimeno A et al. Durvalumab for recurrent or metastatic head and neck squamous cell carcinoma: Results from a single-arm, phase II study in patients with ≥25% tumour cell PD-L1 expression who have progressed on platinum-based chemotherapy. *Eur J Cancer* 2019, 107:142–52. PMID:30576970 doi: 10.1016/j.ejca.2018.11.015
9. [9]. Chan ATC, Lee VHF, Hong RL et al. Pembrolizumab monotherapy versus chemotherapy in platinum-pretreated, recurrent or metastatic nasopharyngeal cancer (KEYNOTE-122): an open-label, randomized, phase III trial. *Ann Oncol* 2023, 34:251–61. PMID:36535566 doi: 10.1016/j.annonc.2022.12.007
10. [10]. Even C, Wang HM, Li SH et al. Phase II, Randomized Study of Spartalizumab (PDR001), an Anti-PD-1 Antibody, versus Chemotherapy in Patients with Recurrent/Metastatic Nasopharyngeal Cancer. *Clin Cancer Res* 2021, 27:6413–23. PMID:34433653 doi: 10.1158/1078-0432.Ccr-21-0822
11. [11]. Chen X, Wang W, Zou Q et al. Penpulimab, an anti-PD-1 antibody, for heavily pretreated metastatic nasopharyngeal carcinoma: a single-arm phase II study. *Signal Transduct Target Ther* 2024, 9:148. PMID:38890298 doi: 10.1038/s41392-024-01865-6
12. [12]. Wang FH, Wei XL, Feng J et al. Efficacy, Safety, and Correlative Biomarkers of Toripalimab in Previously Treated Recurrent or Metastatic Nasopharyngeal Carcinoma: A Phase II Clinical Trial (POLARIS-02). *J Clin Oncol* 2021, 39:704–12. PMID:33492986 doi: 10.1200/jco.20.02712
13. [13]. Demidov L, Kharkevich G, Petenko N et al. A phase III study to access the safety and efficacy of prolgolimab 250 mg fixed dose administered every 3 weeks versus prolgolimab 1 mg/kg every 2 weeks in patients with metastatic melanoma (FLAT). *Front Oncol* 2024, 14:1385685. PMID:39296979 doi: 10.3389/fonc.2024.1385685
14. [14]. Yamazaki N, Kiyohara Y, Uhara H et al. Efficacy and safety of nivolumab in Japanese patients with previously untreated advanced melanoma: A phase II study. *Cancer Sci* 2017, 108:1223–30. PMID:28342215 doi: 10.1111/cas.13241
15. [15]. Maubec E, Boubaya M, Petrow P et al. Phase II Study of Pembrolizumab As First-Line, Single-Drug Therapy for Patients With Unresectable Cutaneous Squamous Cell Carcinomas. *J Clin Oncol* 2020, 38:3051–61. PMID:32730186 doi: 10.1200/jco.19.03357
16. [16]. Lang R, Welponer T, Richtig E et al. Nivolumab for locally advanced and metastatic cutaneous squamous cell carcinoma (NIVOSQUACS study)-Phase II data covering impact of concomitant haematological malignancies. *J Eur Acad Dermatol Venereol* 2023, 37:1799–810. PMID:37210651 doi: 10.1111/jdv.19218

17. [17]. Stratigos AJ, Sekulic A, Peris K et al. Cemiplimab in locally advanced basal cell carcinoma after hedgehog inhibitor therapy: an open-label, multi-centre, single-arm, phase 2 trial. *Lancet Oncol* 2021, 22:848–57. PMID:34000246 doi: 10.1016/s1470-2045(21)00126-1
18. [18]. Mortier L, Villabona L, Lawrence B et al. Pembrolizumab for the First-Line Treatment of Recurrent Locally Advanced or Metastatic Merkel Cell Carcinoma: Results from the Single-Arm, Open-Label, Phase III KEYNOTE-913 Study. *Am J Clin Dermatol* 2024, 25:987–96. PMID:39377880 doi: 10.1007/s40257-024-00885-w
19. [19]. Nghiem P, Bhatia S, Lipson EJ et al. Three-year survival, correlates and salvage therapies in patients receiving first-line pembrolizumab for advanced Merkel cell carcinoma. *J Immunother Cancer* 2021, 9. PMID:33879601 doi: 10.1136/jitc-2021-002478
20. [20]. Kaufman HL, Russell JS, Hamid O et al. Updated efficacy of avelumab in patients with previously treated metastatic Merkel cell carcinoma after ≥1 year of follow-up: JAVELIN Merkel 200, a phase 2 clinical trial. *J Immunother Cancer* 2018, 6:7. PMID:29347993 doi: 10.1186/s40425-017-0310-x
21. [21]. Shitara K, Van Cutsem E, Bang YJ et al. Efficacy and Safety of Pembrolizumab or Pembrolizumab Plus Chemotherapy vs Chemotherapy Alone for Patients With First-line, Advanced Gastric Cancer: The KEYNOTE-062 Phase 3 Randomized Clinical Trial. *JAMA Oncol* 2020, 6:1571–80. PMID:32880601 doi: 10.1001/jamaoncol.2020.3370
22. [22]. Kang YK, Boku N, Satoh T et al. Nivolumab in patients with advanced gastric or gastro-oesophageal junction cancer refractory to, or intolerant of, at least two previous chemotherapy regimens (ONO-4538-12, ATTRACTION-2): a randomised, double-blind, placebo-controlled, phase 3 trial. *Lancet* 2017, 390:2461–71. PMID:28993052 doi: 10.1016/s0140-6736(17)31827-5
23. [23]. Bang YJ, Ruiz EY, Van Cutsem E et al. Phase III, randomised trial of avelumab versus physician's choice of chemotherapy as third-line treatment of patients with advanced gastric or gastro-oesophageal junction cancer: primary analysis of JAVELIN Gastric 300. *Ann Oncol* 2018, 29:2052–60. PMID:30052729 doi: 10.1093/annonc/mdy264
24. [24]. Shitara K, Özgüroğlu M, Bang YJ et al. Pembrolizumab versus paclitaxel for previously treated, advanced gastric or gastro-oesophageal junction cancer (KEYNOTE-061): a randomised, open-label, controlled, phase 3 trial. *Lancet* 2018, 392:123–33. PMID:29880231 doi: 10.1016/s0140-6736(18)31257-1
25. [25]. Shah MA, Cunningham D, Metges JP et al. Randomized, open-label, phase 2 study of andecaliximab plus nivolumab versus nivolumab alone in advanced gastric cancer identifies biomarkers associated with survival. *J Immunother Cancer* 2021, 9. PMID:34893523 doi: 10.1136/jitc-2021-003580
26. [26]. Kim RD, Chung V, Alese OB et al. A Phase 2 Multi-institutional Study of Nivolumab for Patients With Advanced Refractory Biliary Tract Cancer. *JAMA Oncol* 2020, 6:888–94. PMID:32352498 doi: 10.1001/jamaoncol.2020.0930
27. [27]. Yau T, Park JW, Finn RS et al. Nivolumab versus sorafenib in advanced hepatocellular carcinoma (CheckMate 459): a randomised, multicentre, open-label, phase 3 trial. *Lancet Oncol* 2022, 23:77–90. PMID:34914889 doi: 10.1016/s1470-2045(21)00604-5
28. [28]. Qin S, Kudo M, Meyer T et al. Tislelizumab vs Sorafenib as First-Line Treatment for Unresectable Hepatocellular Carcinoma: A Phase 3 Randomized Clinical Trial. *JAMA Oncol* 2023, 9:1651–9. PMID:37796513 doi: 10.1001/jamaoncol.2023.4003
29. [29]. Verset G, Borbath I, Karwal M et al. Pembrolizumab Monotherapy for Previously Untreated Advanced Hepatocellular Carcinoma: Data from the Open-Label, Phase II KEYNOTE-224 Trial. *Clin Cancer Res* 2022, 28:2547–54. PMID:35421228 doi: 10.1158/1078-0432.Ccr-21-3807
30. [30]. Finn RS, Ryoo BY, Merle P et al. Pembrolizumab As Second-Line Therapy in Patients With Advanced Hepatocellular Carcinoma in KEYNOTE-240: A Randomized, Double-Blind, Phase III Trial. *J Clin Oncol* 2020, 38:193–202. PMID:31790344 doi: 10.1200/jco.19.01307
31. [31]. Qin S, Chen Z, Fang W et al. Pembrolizumab Versus Placebo as Second-Line Therapy in Patients From Asia With Advanced Hepatocellular Carcinoma: A Randomized, Double-Blind, Phase III Trial. *J Clin Oncol* 2023, 41:1434–43. PMID:36455168 doi: 10.1200/jco.22.00620
32. [32]. Qin S, Ren Z, Meng Z et al. Camrelizumab in patients with previously treated advanced hepatocellular carcinoma: a multicentre, open-label, parallel-group, randomised, phase 2 trial. *Lancet Oncol* 2020, 21:571–80. PMID:32112738 doi: 10.1016/s1470-2045(20)30011-5

33. [33]. Kudo M, Finn RS, Edeline J et al. Updated efficacy and safety of KEYNOTE-224: a phase II study of pembrolizumab in patients with advanced hepatocellular carcinoma previously treated with sorafenib. *Eur J Cancer* 2022, 167:1–12. PMID:35364421 doi: 10.1016/j.ejca.2022.02.009
34. [34]. Ren Z, Ducreux M, Abou-Alfa GK et al. Tislelizumab in Patients with Previously Treated Advanced Hepatocellular Carcinoma (RATIONALE-208): A Multicenter, Non-Randomized, Open-Label, Phase 2 Trial. *Liver Cancer* 2023, 12:72–84. PMID:36872927 doi: 10.1159/000527175
35. [35]. André T, Shiu KK, Kim TW et al. Pembrolizumab versus chemotherapy in microsatellite instability-high or mismatch repair-deficient metastatic colorectal cancer: 5-year follow-up from the randomized phase III KEYNOTE-177 study. *Ann Oncol* 2025, 36:277–84. PMID:39631622 doi: 10.1016/j.annonc.2024.11.012
36. [36]. Overman MJ, McDermott R, Leach JL et al. Nivolumab in patients with metastatic DNA mismatch repair-deficient or microsatellite instability-high colorectal cancer (CheckMate 142): an open-label, multicentre, phase 2 study. *Lancet Oncol* 2017, 18:1182–91. PMID:28734759 doi: 10.1016/s1470-2045(17)30422-9
37. [37]. Oh CR, Kim JE, Hong YS et al. Phase II study of durvalumab monotherapy in patients with previously treated microsatellite instability-high/mismatch repair-deficient or POLE-mutated metastatic or unresectable colorectal cancer. *Int J Cancer* 2022, 150:2038–45. PMID:35179785 doi: 10.1002/ijc.33966
38. [38]. Le DT, Diaz LA, Jr., Kim TW et al. Pembrolizumab for previously treated, microsatellite instability-high/mismatch repair-deficient advanced colorectal cancer: final analysis of KEYNOTE-164. *Eur J Cancer* 2023, 186:185–95. PMID:37141828 doi: 10.1016/j.ejca.2023.02.016
39. [39]. Marabelle A, Cassier PA, Fakih M et al. Pembrolizumab for previously treated advanced anal squamous cell carcinoma: results from the non-randomised, multicohort, multicentre, phase 2 KEYNOTE-158 study. *Lancet Gastroenterol Hepatol* 2022, 7:446–54. PMID:35114169 doi: 10.1016/s2468-1253(21)00382-4
40. [40]. Rao S, Anandappa G, Capdevila J et al. A phase II study of retifanlimab (INCMGA00012) in patients with squamous carcinoma of the anal canal who have progressed following platinum-based chemotherapy (POD1UM-202). *ESMO Open* 2022, 7:100529. PMID:35816951 doi: 10.1016/j.esmoop.2022.100529
41. [41]. Morris VK, Salem ME, Nimeiri H et al. Nivolumab for previously treated unresectable metastatic anal cancer (NCI9673): a multicentre, single-arm, phase 2 study. *Lancet Oncol* 2017, 18:446–53. PMID:28223062 doi: 10.1016/s1470-2045(17)30104-3
42. [42]. Kojima T, Shah MA, Muro K et al. Randomized Phase III KEYNOTE-181 Study of Pembrolizumab Versus Chemotherapy in Advanced Esophageal Cancer. *J Clin Oncol* 2020, 38:4138–48. PMID:33026938 doi: 10.1200/jco.20.01888
43. [43]. Shen L, Kato K, Kim SB et al. Tislelizumab Versus Chemotherapy as Second-Line Treatment for Advanced or Metastatic Esophageal Squamous Cell Carcinoma (RATIONALE-302): A Randomized Phase III Study. *J Clin Oncol* 2022, 40:3065–76. PMID:35442766 doi: 10.1200/jco.21.01926
44. [44]. Huang J, Xu J, Chen Y et al. Camrelizumab versus investigator's choice of chemotherapy as second-line therapy for advanced or metastatic oesophageal squamous cell carcinoma (ESCORT): a multicentre, randomised, open-label, phase 3 study. *Lancet Oncol* 2020, 21:832–42. PMID:32416073 doi: 10.1016/s1470-2045(20)30110-8
45. [45]. Kato K, Cho BC, Takahashi M et al. Nivolumab versus chemotherapy in patients with advanced oesophageal squamous cell carcinoma refractory or intolerant to previous chemotherapy (ATTRACTION-3): a multicentre, randomised, open-label, phase 3 trial. *Lancet Oncol* 2019, 20:1506–17. PMID:31582355 doi: 10.1016/s1470-2045(19)30626-6
46. [46]. Janjigian YY, Bendell J, Calvo E et al. CheckMate-032 Study: Efficacy and Safety of Nivolumab and Nivolumab Plus Ipilimumab in Patients With Metastatic Esophagogastric Cancer. *J Clin Oncol* 2018, 36:2836–44. PMID:30110194 doi: 10.1200/jco.2017.76.6212
47. [47]. Shah MA, Kojima T, Hochhauser D et al. Efficacy and Safety of Pembrolizumab for Heavily Pretreated Patients With Advanced, Metastatic Adenocarcinoma or Squamous Cell Carcinoma of the Esophagus: The Phase 2 KEYNOTE-180 Study. *JAMA Oncol* 2019, 5:546–50. PMID:30570649 doi: 10.1001/jamaoncol.2018.5441
48. [48]. Xu J, Li Y, Fan Q et al. Clinical and biomarker analyses of sintilimab versus chemotherapy as second-line therapy for advanced or metastatic esophageal squamous cell carcinoma: a randomized, open-label phase 2 study (ORIENT-2). *Nat Commun* 2022, 13:857. PMID:35165274 doi: 10.1038/s41467-022-28408-3

49. [49].de Klerk LK, Patel AK, Derks S et al. Phase II study of pembrolizumab in refractory esophageal cancer with correlates of response and survival. *J Immunother Cancer* 2021, 9. PMID:34593617 doi: 10.1136/jitc-2021-002472
50. [50].Kudo T, Hamamoto Y, Kato K et al. Nivolumab treatment for oesophageal squamous-cell carcinoma: an open-label, multicentre, phase 2 trial. *Lancet Oncol* 2017, 18:631–9. PMID:28314688 doi: 10.1016/s1470-2045(17)30181-x
51. [51].Canova S, Ceresoli GL, Grosso F et al. Final results of DIADEM, a phase II study to investigate the efficacy and safety of durvalumab in advanced pretreated malignant pleural mesothelioma. *ESMO Open* 2022, 7:100644. PMID:36463732 doi: 10.1016/j.esmoop.2022.100644
52. [52].Fujimoto N, Okada M, Kijima T et al. Clinical Efficacy and Safety of Nivolumab in Japanese Patients With Malignant Pleural Mesothelioma: 3-Year Results of the MERIT Study. *JTO Clin Res Rep* 2021, 2:100135. PMID:34589998 doi: 10.1016/j.jtocrr.2020.100135
53. [53].Giaccone G, Kim C, Thompson J et al. Pembrolizumab in patients with thymic carcinoma: a single-arm, single-centre, phase 2 study. *Lancet Oncol* 2018, 19:347–55. PMID:29395863 doi: 10.1016/s1470-2045(18)30062-7
54. [54].Cho J, Kim HS, Ku BM et al. Pembrolizumab for Patients With Refractory or Relapsed Thymic Epithelial Tumor: An Open-Label Phase II Trial. *J Clin Oncol* 2019, 37:2162–70. PMID:29906252 doi: 10.1200/jco.2017.77.3184
55. [55].Lu S, Ma X, Liu L et al. Efficacy and Safety of Atezolizumab in Chinese Patients With Advanced Thymic Carcinoma: A Multicenter, Single-Arm Phase 2 Study. *Clin Lung Cancer* 2025, 26:244–52.e2. PMID:39986946 doi: 10.1016/j.cllc.2025.01.011
56. [56].Katsuya Y, Horinouchi H, Seto T et al. Single-arm, multicentre, phase II trial of nivolumab for unresectable or recurrent thymic carcinoma: PRIMER study. *Eur J Cancer* 2019, 113:78–86. PMID:30991261 doi: 10.1016/j.ejca.2019.03.012
57. [57].Pujol JL, Greillier L, Audigier-Valette C et al. A Randomized Non-Comparative Phase II Study of Anti-Programmed Cell Death-Ligand 1 Atezolizumab or Chemotherapy as Second-Line Therapy in Patients With Small Cell Lung Cancer: Results From the IFCT-1603 Trial. *J Thorac Oncol* 2019, 14:903–13. PMID:30664989 doi: 10.1016/j.jtho.2019.01.008
58. [58].Carbone DP, Reck M, Paz-Ares L et al. First-Line Nivolumab in Stage IV or Recurrent Non-Small-Cell Lung Cancer. *N Engl J Med* 2017, 376:2415–26. PMID:28636851 doi: 10.1056/NEJMoa1613493
59. [59].Reck M, Rodríguez-Abreu D, Robinson AG et al. Pembrolizumab versus Chemotherapy for PD-L1-Positive Non-Small-Cell Lung Cancer. *N Engl J Med* 2016, 375:1823–33. PMID:27718847 doi: 10.1056/NEJMoa1606774
60. [60].Sezer A, Kilickap S, Gümüş M et al. Cemiplimab monotherapy for first-line treatment of advanced non-small-cell lung cancer with PD-L1 of at least 50%: a multicentre, open-label, global, phase 3, randomised, controlled trial. *Lancet* 2021, 397:592–604. PMID:33581821 doi: 10.1016/s0140-6736(21)00228-2
61. [61].de Castro G, Jr., Kudaba I, Wu YL et al. Five-Year Outcomes With Pembrolizumab Versus Chemotherapy as First-Line Therapy in Patients With Non-Small-Cell Lung Cancer and Programmed Death Ligand-1 Tumor Proportion Score ≥ 1% in the KEYNOTE-042 Study. *J Clin Oncol* 2023, 41:1986–91. PMID:36306479 doi: 10.1200/jco.21.02885
62. [62].Lee SM, Schulz C, Prabhash K et al. First-line atezolizumab monotherapy versus single-agent chemotherapy in patients with non-small-cell lung cancer ineligible for treatment with a platinum-containing regimen (IPSOS): a phase 3, global, multicentre, open-label, randomised controlled study. *Lancet* 2023, 402:451–63. PMID:37423228 doi: 10.1016/s0140-6736(23)00774-2
63. [63].Reck M, Barlesi F, Yang JC et al. Avelumab Versus Platinum-Based Doublet Chemotherapy as First-Line Treatment for Patients With High-Expression Programmed Death-Ligand 1-Positive Metastatic NSCLC: Primary Analysis From the Phase 3 JAVELIN Lung 100 Trial. *J Thorac Oncol* 2024, 19:297–313. PMID:37748693 doi: 10.1016/j.jtho.2023.09.1445
64. [64].Shaverdashvili K, Reyes V, Wang H et al. A phase II clinical trial evaluating the safety and efficacy of durvalumab as first line therapy in advanced and metastatic non-small cell lung cancer patients with Eastern Cooperative Oncology Group performance status of 2. *EClinicalMedicine* 2023, 66:102317. PMID:38192592 doi: 10.1016/j.eclinm.2023.102317
65. [65].Tokito T, Kolesnik O, Sørensen J et al. Epacadostat plus pembrolizumab versus placebo plus pembrolizumab as first-line treatment for metastatic non-small cell lung cancer with high levels of programmed death-ligand 1: a randomized, double-blind phase 2 study. *BMC Cancer* 2024, 23:1251. PMID:39054476 doi: 10.1186/s12885-023-11203-8
66. [66].Di Giacomo AM, Schenker M, Medioni J et al. A phase II study of retifanlimab, a humanized anti-PD-1 monoclonal antibody, in patients with solid tumors (POD1UM-203). *ESMO Open* 2024, 9:102387. PMID:38401247 doi: 10.1016/j.esmoop.2024.102387

67. [67].Maggie Liu SY, Huang J, Deng JY et al. PD-L1 expression guidance on sintilimab versus pembrolizumab with or without platinum-doublet chemotherapy in untreated patients with advanced non-small cell lung cancer (CTONG1901): A phase 2, randomized, controlled trial. *Sci Bull (Beijing)* 2024, 69:535–43. PMID:38185589 doi: 10.1016/j.scib.2023.12.046
68. [68].Mark M, Froesch P, Gysel K et al. First-line durvalumab in patients with PD-L1 positive, advanced non-small cell lung cancer (NSCLC) with a performance status of 2 (PS2). Primary analysis of the multicenter, single-arm phase II trial SAKK 19/17. *Eur J Cancer* 2024, 200:113600. PMID:38330766 doi: 10.1016/j.ejca.2024.113600
69. [69].Peters S, Gettinger S, Johnson ML et al. Phase II Trial of Atezolizumab As First-Line or Subsequent Therapy for Patients With Programmed Death-Ligand 1-Selected Advanced Non-Small-Cell Lung Cancer (BIRCH). *J Clin Oncol* 2017, 35:2781–9. PMID:28609226 doi: 10.1200/jco.2016.71.9476
70. [70].Fehrenbacher L, von Pawel J, Park K et al. Updated Efficacy Analysis Including Secondary Population Results for OAK: A Randomized Phase III Study of Atezolizumab versus Docetaxel in Patients with Previously Treated Advanced Non-Small Cell Lung Cancer. *J Thorac Oncol* 2018, 13:1156–70. PMID:29777823 doi: 10.1016/j.jtho.2018.04.039
71. [71].Ardizzoni A, Azevedo S, Rubio-Viqueira B et al. Primary results from TAIL: a global single-arm safety study of atezolizumab monotherapy in a diverse population of patients with previously treated advanced non-small cell lung cancer. *J Immunother Cancer* 2021, 9. PMID:33737339 doi: 10.1136/jitc-2020-001865
72. [72].Borghaei H, Paz-Ares L, Horn L et al. Nivolumab versus Docetaxel in Advanced Nonsquamous Non-Small-Cell Lung Cancer. *N Engl J Med* 2015, 373:1627–39. PMID:26412456 doi: 10.1056/NEJMoa1507643
73. [73].Brahmer J, Reckamp KL, Baas P et al. Nivolumab versus Docetaxel in Advanced Squamous-Cell Non-Small-Cell Lung Cancer. *N Engl J Med* 2015, 373:123–35. PMID:26028407 doi: 10.1056/NEJMoa1504627
74. [74].Lu S, Cheng Y, Zhou J et al. An open label, safety study of Asian patients with advanced non-small-cell lung cancer receiving second-line nivolumab monotherapy (CheckMate 870). *Ther Adv Med Oncol* 2022, 14:17588359221138380. PMID:36425873 doi: 10.1177/17588359221138380
75. [75].Lu S, Wang J, Cheng Y et al. Nivolumab versus docetaxel in a predominantly Chinese patient population with previously treated advanced non-small cell lung cancer: 2-year follow-up from a randomized, open-label, phase 3 study (CheckMate 078). *Lung Cancer* 2021, 152:7–14. PMID:33321441 doi: 10.1016/j.lungcan.2020.11.013
76. [76].Shi Y, Wu L, Yu X et al. Sintilimab versus docetaxel as second-line treatment in advanced or metastatic squamous non-small-cell lung cancer: an open-label, randomized controlled phase 3 trial (ORIENT-3). *Cancer Commun (Lond)* 2022, 42:1314–30. PMID:36336841 doi: 10.1002/cac2.12385
77. [77].Zhou C, Huang D, Fan Y et al. Tislelizumab Versus Docetaxel in Patients With Previously Treated Advanced NSCLC (RATIONALE-303): A Phase 3, Open-Label, Randomized Controlled Trial. *J Thorac Oncol* 2023, 18:93–105. PMID:36184068 doi: 10.1016/j.jtho.2022.09.217
78. [78].Ren S, Feng J, Ma S et al. KEYNOTE-033: Randomized phase 3 study of pembrolizumab vs docetaxel in previously treated, PD-L1-positive, advanced NSCLC. *Int J Cancer* 2023, 153:623–34. PMID:37141294 doi: 10.1002/ijc.34532
79. [79].Park K, Özgüroğlu M, Vansteenkiste J et al. Avelumab Versus Docetaxel in Patients With Platinum-Treated Advanced NSCLC: 2-Year Follow-Up From the JAVELIN Lung 200 Phase 3 Trial. *J Thorac Oncol* 2021, 16:1369–78. PMID:33845211 doi: 10.1016/j.jtho.2021.03.009
80. [80].Lee JS, Lee KH, Cho EK et al. Nivolumab in advanced non-small-cell lung cancer patients who failed prior platinum-based chemotherapy. *Lung Cancer* 2018, 122:234–42. PMID:30032838 doi: 10.1016/j.lungcan.2018.05.023
81. [81].Borghaei H, Redman MW, Kelly K et al. SWOG S1400A (NCT02154490): A Phase II Study of Durvalumab for Patients With Previously Treated Stage IV or Recurrent Squamous Cell Lung Cancer (Lung-MAP Sub-study). *Clin Lung Cancer* 2021, 22:178–86. PMID:33358401 doi: 10.1016/j.cllc.2020.10.015
82. [82].Felip E, Ardizzoni A, Ciuleanu T et al. CheckMate 171: A phase 2 trial of nivolumab in patients with previously treated advanced squamous non-small cell lung cancer, including ECOG PS 2 and elderly populations. *Eur J Cancer* 2020, 127:160–72. PMID:32028209 doi: 10.1016/j.ejca.2019.11.019
83. [83].Chen YM, Chih-Hsin Yang J, Su WC et al. Nivolumab safety and efficacy in advanced, platinum-resistant, non-small cell lung cancer, radical radiotherapy-ineligible patients: A phase II study in Taiwan. *J Formos Med Assoc* 2020, 119:1817–26. PMID:32094063 doi: 10.1016/j.jfma.2020.01.004

84. [84]. Yang JJ, Huang C, Fan Y et al. Camrelizumab in different PD-L1 expression cohorts of pre-treated advanced or metastatic non-small cell lung cancer: a phase II study. *Cancer Immunol Immunother* 2022, 71:1393–402. PMID:34668977 doi: 10.1007/s00262-021-03091-3
85. [85]. Wang Y, Hao Q, Nie J et al. Nivolumab combined docetaxel versus nivolumab in patients with previously treated nonsmall cell lung cancer: a phase 2 study. *Anticancer Drugs* 2024, 35:412–7. PMID:38240789 doi: 10.1097/cad.0000000000001569
86. [86]. Hida T, Nishio M, Nogami N et al. Efficacy and safety of nivolumab in Japanese patients with advanced or recurrent squamous non-small cell lung cancer. *Cancer Sci* 2017, 108:1000–6. PMID:28266091 doi: 10.1111/cas.13225
87. [87]. Nishio M, Hida T, Atagi S et al. Multicentre phase II study of nivolumab in Japanese patients with advanced or recurrent non-squamous non-small cell lung cancer. *ESMO Open* 2016, 1:e000108. PMID:28861280 doi: 10.1136/esmoopen-2016-000108
88. [88]. Akamatsu H, Teraoka S, Takamori S et al. Nivolumab Retreatment in Non-Small Cell Lung Cancer Patients Who Responded to Prior Immune Checkpoint Inhibitors and Had ICI-Free Intervals (WJOG9616L). *Clin Cancer Res* 2022, 28:Of1–of7. PMID:35762926 doi: 10.1158/1078-0432.Ccr-22-0602
89. [89]. McDermott DF, Lee JL, Bjarnason GA et al. Open-Label, Single-Arm Phase II Study of Pembrolizumab Monotherapy as First-Line Therapy in Patients With Advanced Clear Cell Renal Cell Carcinoma. *J Clin Oncol* 2021, 39:1020–8. PMID:33529051 doi: 10.1200/jco.20.02363
90. [90]. McDermott DF, Lee JL, Ziobro M et al. Open-Label, Single-Arm, Phase II Study of Pembrolizumab Monotherapy as First-Line Therapy in Patients With Advanced Non-Clear Cell Renal Cell Carcinoma. *J Clin Oncol* 2021, 39:1029–39. PMID:33529058 doi: 10.1200/jco.20.02365
91. [91]. Atkins MB, Jegede OA, Haas NB et al. Phase II Study of Nivolumab and Salvage Nivolumab/Ipilimumab in Treatment-Naive Patients With Advanced Clear Cell Renal Cell Carcinoma (HCRN GU16-260-Cohort A). *J Clin Oncol* 2022, 40:2913–23. PMID:35442713 doi: 10.1200/jco.21.02938
92. [92]. Atkins MB, Jegede OA, Haas NB et al. Phase II study of nivolumab and salvage nivolumab/ipilimumab in treatment-naïve patients with advanced non-clear cell renal cell carcinoma (HCRN GU16-260-Cohort B). *J Immunother Cancer* 2023, 11. PMID:36948504 doi: 10.1136/jitc-2022-004780
93. [93]. Motzer RJ, Escudier B, George S et al. Nivolumab versus everolimus in patients with advanced renal cell carcinoma: Updated results with long-term follow-up of the randomized, open-label, phase 3 CheckMate 025 trial. *Cancer* 2020, 126:4156–67. PMID:32673417 doi: 10.1002/cncr.33033
94. [94]. Motzer RJ, Rini BI, McDermott DF et al. Nivolumab for Metastatic Renal Cell Carcinoma: Results of a Randomized Phase II Trial. *J Clin Oncol* 2015, 33:1430–7. PMID:25452452 doi: 10.1200/jco.2014.59.0703
95. [95]. Voss MH, Azad AA, Hansen AR et al. A Randomized Phase II Study of MEDI0680 in Combination with Durvalumab versus Nivolumab Monotherapy in Patients with Advanced or Metastatic Clear-cell Renal Cell Carcinoma. *Clin Cancer Res* 2022, 28:3032–41. PMID:35507017 doi: 10.1158/1078-0432.Ccr-21-4115
96. [96]. Raj N, Zheng Y, Kelly V et al. PD-1 Blockade in Advanced Adrenocortical Carcinoma. *J Clin Oncol* 2020, 38:71–80. PMID:31644329 doi: 10.1200/jco.19.01586
97. [97]. Carneiro BA, Konda B, Costa RB et al. Nivolumab in Metastatic Adrenocortical Carcinoma: Results of a Phase 2 Trial. *J Clin Endocrinol Metab* 2019, 104:6193–200. PMID:31276163 doi: 10.1210/jc.2019-00600
98. [98]. Galsky MD, Arija JÁ A, Bamias A et al. Atezolizumab with or without chemotherapy in metastatic urothelial cancer (IMvigor130): a multicentre, randomised, placebo-controlled phase 3 trial. *Lancet* 2020, 395:1547–57. PMID:32416780 doi: 10.1016/s0140-6736(20)30230-0
99. [99]. Powles T, Csőszi T, Özgüroğlu M et al. Pembrolizumab alone or combined with chemotherapy versus chemotherapy as first-line therapy for advanced urothelial carcinoma (KEYNOTE-361): a randomised, open-label, phase 3 trial. *Lancet Oncol* 2021, 22:931–45. PMID:34051178 doi: 10.1016/s1470-2045(21)00152-2
100. [100]. Vuky J, Balar AV, Castellano D et al. Long-Term Outcomes in KEYNOTE-052: Phase II Study Investigating First-Line Pembrolizumab in Cisplatin-Ineligible Patients With Locally Advanced or Metastatic Urothelial Cancer. *J Clin Oncol* 2020, 38:2658–66. PMID:32552471 doi: 10.1200/jco.19.01213

101. [101]. Rosenberg JE, Galsky MD, Powles T et al. Atezolizumab monotherapy for metastatic urothelial carcinoma: final analysis from the phase II IMvigor210 trial. *ESMO Open* 2024, 9:103972. PMID:39642637 doi: 10.1016/j.esmoop.2024.103972
102. [102]. Fradet Y, Bellmunt J, Vaughn DJ et al. Randomized phase III KEYNOTE-045 trial of pembrolizumab versus paclitaxel, docetaxel, or vinflunine in recurrent advanced urothelial cancer: results of >2 years of follow-up. *Ann Oncol* 2019, 30:970–6. PMID:31050707 doi: 10.1093/annonc/mdz127
103. [103]. Powles T, Durán I, van der Heijden MS et al. Atezolizumab versus chemotherapy in patients with platinum-treated locally advanced or metastatic urothelial carcinoma (IMvigor211): a multicentre, open-label, phase 3 randomised controlled trial. *Lancet* 2018, 391:748–57. PMID:29268948 doi: 10.1016/s0140-6736(17)33297-x
104. [104]. Sternberg CN, Loriot Y, James N et al. Primary Results from SAUL, a Multinational Single-arm Safety Study of Atezolizumab Therapy for Locally Advanced or Metastatic Urothelial or Nonurothelial Carcinoma of the Urinary Tract. *Eur Urol* 2019, 76:73–81. PMID:30910346 doi: 10.1016/j.eururo.2019.03.015
105. [105]. Sharma P, Retz M, Siefker-Radtke A et al. Nivolumab in metastatic urothelial carcinoma after platinum therapy (CheckMate 275): a multicentre, single-arm, phase 2 trial. *Lancet Oncol* 2017, 18:312–22. PMID:28131785 doi: 10.1016/s1470-2045(17)30065-7
106. [106]. Zhang T, Harrison MR, O'Donnell PH et al. A randomized phase 2 trial of pembrolizumab versus pembrolizumab and acalabrutinib in patients with platinum-resistant metastatic urothelial cancer. *Cancer* 2020, 126:4485–97. PMID:32757302 doi: 10.1002/cncr.33067
107. [107]. Ye D, Liu J, Zhou A et al. Tislelizumab in Asian patients with previously treated locally advanced or metastatic urothelial carcinoma. *Cancer Sci* 2021, 112:305–13. PMID:33047430 doi: 10.1111/cas.14681
108. [108]. Sheng X, Chen H, Hu B et al. Safety, Efficacy, and Biomarker Analysis of Toripalimab in Patients with Previously Treated Advanced Urothelial Carcinoma: Results from a Multicenter Phase II Trial POLARIS-03. *Clin Cancer Res* 2022, 28:489–97. PMID:34740921 doi: 10.1158/1078-0432.Ccr-21-2210
109. [109]. Marabelle A, Le DT, Ascierto PA et al. Efficacy of Pembrolizumab in Patients With Noncolorectal High Microsatellite Instability/Mismatch Repair-Deficient Cancer: Results From the Phase II KEYNOTE-158 Study. *J Clin Oncol* 2020, 38:1–10. PMID:31682550 doi: 10.1200/jco.19.02105
110. [110]. Zeverijn LJ, Geurts BS, Battaglia TW et al. The Innate Immune Landscape of dMMR/MSI Cancers Predicts the Outcome of Nivolumab Treatment: Results from the Drug Rediscovery Protocol. *Clin Cancer Res* 2024, 30:4339–51. PMID:39024037 doi: 10.1158/1078-0432.Ccr-24-0480
111. [111]. de Vries HM, Rafael TS, Gil-Jimenez A et al. Atezolizumab With or Without Radiotherapy for Advanced Squamous Cell Carcinoma of the Penis (The PERICLES Study): A Phase II Trial. *J Clin Oncol* 2023, 41:4872–80. PMID:37487169 doi: 10.1200/jco.22.02894
112. [112]. García Del Muro X, Páez López-Bravo D, Cuéllar-Rivas MA et al. Retifanlimab in Advanced Penile Squamous Cell Carcinoma: The Phase 2 ORPHEUS Study. *Eur Urol Oncol* 2025, 8:278–86. PMID:38749903 doi: 10.1016/j.euo.2024.04.021
113. [113]. Antonarakis ES, Piulats JM, Gross-Goupil M et al. Pembrolizumab for Treatment-Refractory Metastatic Castration-Resistant Prostate Cancer: Multicohort, Open-Label Phase II KEYNOTE-199 Study. *J Clin Oncol* 2020, 38:395–405. PMID:31774688 doi: 10.1200/jco.19.01638
114. [114]. Brown LC, Halabi S, Somarelli JA et al. A phase 2 trial of avelumab in men with aggressive-variant or neuroendocrine prostate cancer. *Prostate Cancer Prostatic Dis* 2022, 25:762–9. PMID:35292724 doi: 10.1038/s41391-022-00524-7
115. [115]. Adams S, Schmid P, Rugo HS et al. Pembrolizumab monotherapy for previously treated metastatic triple-negative breast cancer: cohort A of the phase II KEYNOTE-086 study. *Ann Oncol* 2019, 30:397–404. PMID:30475950 doi: 10.1093/annonc/mdy517
116. [116]. O'Malley DM, Bariani GM, Cassier PA et al. Pembrolizumab in Patients With Microsatellite Instability-High Advanced Endometrial Cancer: Results From the KEYNOTE-158 Study. *J Clin Oncol* 2022, 40:752–61. PMID:34990208 doi: 10.1200/jco.21.01874

117. [117]. Chung HC, Ros W, Delord JP et al. Efficacy and Safety of Pembrolizumab in Previously Treated Advanced Cervical Cancer: Results From the Phase II KEYNOTE-158 Study. *J Clin Oncol* 2019, 37:1470–8. PMID:30943124 doi: 10.1200/jco.18.01265
118. [118]. O'Malley DM, Oaknin A, Monk BJ et al. Phase II study of the safety and efficacy of the anti-PD-1 antibody balstilimab in patients with recurrent and/or metastatic cervical cancer. *Gynecol Oncol* 2021, 163:274–80. PMID:34452745 doi: 10.1016/j.ygyno.2021.08.018
119. [119]. Tewari KS, Monk BJ, Vergote I et al. Survival with Cemiplimab in Recurrent Cervical Cancer. *N Engl J Med* 2022, 386:544–55. PMID:35139273 doi: 10.1056/NEJMoa2112187
120. [120]. Xia L, Wang J, Wang C et al. Efficacy and safety of zimberelimab (GLS-010) monotherapy in patients with recurrent or metastatic cervical cancer: a multicenter, single-arm, phase II study. *Int J Gynecol Cancer* 2023, 33:1861–8. PMID:37875323 doi: 10.1136/ijgc-2023-004705
121. [121]. Salani R, McCormack M, Kim YM et al. A non-comparative, randomized, phase II trial of atezolizumab or atezolizumab plus tiragolumab for programmed death-ligand 1-positive recurrent cervical cancer (SKYSCRAPER-04). *Int J Gynecol Cancer* 2024, 34:1140–8. PMID:38858106 doi: 10.1136/ijgc-2024-005588
122. [122]. Pujade-Lauraine E, Fujiwara K, Ledermann JA et al. Avelumab alone or in combination with chemotherapy versus chemotherapy alone in platinum-resistant or platinum-refractory ovarian cancer (JAVELIN Ovarian 200): an open-label, three-arm, randomised, phase 3 study. *Lancet Oncol* 2021, 22:1034–46. PMID:34143970 doi: 10.1016/s1470-2045(21)00216-3
123. [123]. Hamanishi J, Takeshima N, Katsumata N et al. Nivolumab Versus Gemcitabine or Pegylated Liposomal Doxorubicin for Patients With Platinum-Resistant Ovarian Cancer: Open-Label, Randomized Trial in Japan (NINJA). *J Clin Oncol* 2021, 39:3671–81. PMID:34473544 doi: 10.1200/jco.21.00334
124. [124]. Matulonis UA, Shapira-Frommer R, Santin AD et al. Antitumor activity and safety of pembrolizumab in patients with advanced recurrent ovarian cancer: results from the phase II KEYNOTE-100 study. *Ann Oncol* 2019, 30:1080–7. PMID:31046082 doi: 10.1093/annonc/mdz135
